# The dual blockade of MET and VEGFR2 signaling demonstrates pronounced inhibition on tumor growth and metastasis of hepatocellular carcinoma

**DOI:** 10.1186/s13046-018-0750-2

**Published:** 2018-04-30

**Authors:** Yu Zhang, Xiaomei Gao, Ying Zhu, Dhruba Kadel, Haoran Sun, Jing Chen, Qin Luo, Haoting Sun, Luyu Yang, Jing Yang, Yuanyuan Sheng, Yan Zheng, Kejin Zhu, Qiongzhu Dong, Lunxiu Qin

**Affiliations:** 1 Department of General Surgery, Huashan Hospital, Cancer Metastasis Institute and Institutes of Biomedical Sciences, Fudan University, 12 Urumqi Road (M), Shanghai, 200040 China; 2 Department of Pharmacology and Cancer Biology, Duke University School of Medicine, Duke University, Durham, NC USA; 3 Duke Cancer Institute, Duke University, Durham, NC USA; 4 Kanion Research Institute, 58 Kangyuan Road, Lianyungang, 222002 Jiangsu China

**Keywords:** Hepatocellular carcinoma, MET, VEGFR2, Molecular targeted blockade, Metastasis

## Abstract

**Background:**

The application of VEGF signaling inhibitors have been associated with more invasive or metastatic behavior of cancers including hepatocellular carcinoma (HCC). We explored the contribution of MET pathway to the enhanced HCC invasion and metastasis by VEGF signaling inhibition, and investigated the antitumor effects of NZ001, a novel dual inhibitor of MET and VEGFR2, in HCC.

**Methods:**

Immunocompetent orthotopic mice model of hepal-6 was established to investigate the effects of either VEGF antibody alone or in combination with the selective MET inhibitor on tumor aggressiveness. The antitumor effects of NZ001 were examined in cultured HCC cells as well as in vivo models. MET gene amplification was determined by SNP 6.0 assay. MET/P-MET expression was detected by IHC.

**Results:**

Selective VEGF signaling inhibition by VEGF antibody significantly reduced in vivo tumor growth of the orthotopic mice models, simultaneously also enhanced tumor invasion and metastasis, but inhibiting MET signaling attenuated this side-effect. Further study revealed that hypoxia caused by VEGF signaling inhibition induced HIF-1α nuclear accumulation, subsequently leading to elevated total-MET expression, and synergized with HGF in inducing invasion. NZ001, a novel dual inhibitor of MET and VEGFR2, markedly inhibited both tumor growth and metastasis of HCC, which showed obvious advantages over sorafenib in not inducing more invasive and metastatic behaviors. This effect is more pronounced in HCC with MET amplification and overexpression.

**Conclusions:**

The activation of MET is responsible for the metastasis-promoting effects induced by VEGF inhibition. MET and VEGFR2 dual blockade, NZ001, has advantages over sorafenib in not inducing more invasive and metastatic behaviors; MET amplification and overexpression can be used to identify the subgroup of patients most likely to get the optimal benefit from NZ001 treatment.

**Electronic supplementary material:**

The online version of this article (10.1186/s13046-018-0750-2) contains supplementary material, which is available to authorized users.

## Background

Hepatocellular carcinoma (HCC) is the sixth most common cancer and one of the leading causes of cancer-related deaths in the world [1]. HCC is an aggressive cancer with a very dismal prognosis due to a high incidence of metastasis at diagnosis and lack of effective medicinal treatment. Induction of angiogenesis has been recognized as a crucial step and one hallmark of cancer progression [2]. Given the key importance of VEGF and its receptor VEGFR in angiogenesis, hopes were raised that blocking this pathway would eradicate the tumor vasculature and provide cancer patients maximal survival benefit. Currently sorafenib, a VEGFR inhibitor which also counters the activity of platelet-derived growth factor receptor β (PDGFR-β), the cytokine receptor c-KIT, Raf-1 and B-Raf, is the first line treatment that was approved by the U.S. Food and Drug Administration (FDA) for the advanced-stage HCC patients [3, 4]. However, the survival benefit of sorafenib is limited, and preclinical studies have shown that the initial suppression of tumor vasculature and tumor growth by VEGFR inhibitor treatments (such as sorafenib and sunitinib) succumbs to rapid revascularization and leads to more invasive and metastatic behavior of cancers [5, 6]. So, it is urgent to explore the involved mechanisms and develop novel strategies to enhance their efficacy and neutralize the side-effects on cancer invasion and metastasis.

MET, a transmembrane tyrosine kinase receptor for hepatocyte growth factor (HGF), has been observed to contribute to cancer metastasis, resistance to chemotherapeutic agents, and dismal outcomes of patients with solid cancers including HCC [7–10]. Within tumor environments, VEGFR and MET signaling pathways have synergistic effects on tumor growth [11, 12]. Emerging evidences suggest that HGF/MET pathway plays important role in the development of resistance to antiangiogenic therapy [13]. Therefore, dual inhibition of MET and VEGF pathways may critically disrupt angiogenesis, tumorigenesis and progression of cancers. Although multiple therapies targeting the MET and VEGFR2 pathways have been described to have clinical benefits in HCC treatment [14, 15], it is unclear whether simultaneous inhibition of MET and VEGFR2 signaling is necessary and sufficient to inhibit HCC invasiveness and metastasis.

In the present study, first we identified the contribution of MET signaling induced by inhibition of VEGF signaling to promote malignancy of HCC in preclinical models. Then we tested if NZ001, a novel ATP-competitive multi-targeted kinase inhibitor that simultaneously inhibits both MET and VEGFR2, could suppress both tumor growth and metastasis. Finally, we found MET amplification and overexpression were useful in subgrouping the HCC patients that might get the optimal benefit from MET inhibitor treatment.

## Methods

### Reagents and antibodies

For in vitro assays, NZ001 was obtained from Nanjing Zhongrunyuan Pharmaceutical Company (Nanjing, Jiangsu, China). XL184, sorafenib and PF-04217903 were purchased from Selleck Chemicals (Houston, TX, USA). Goat anti-mouse VEGF antibody (AF-493-NA) was purchased from R&D Systems (Minneapolis, MN, USA). NZ001, XL184 and PF-04217903 were prepared as a 20-mM stock solution in DMSO (Sigma-Aldrich, St. Louis, USA) for in vitro studies. For in vivo studies, NZ001 was formulated in sterile ddH2O and administered via oral gavage at 10 mg/kg or 30 mg/kg. Sorafenib was dissolved in Cremophor EL/ethanol (50:50; Sigma Cremophor EL, 95% ethyl alcohol) at 4-fold (4×) the highest dose. The final dosing solutions were prepared on the day of use by diluting to 1× with ddH2O and were administered via oral gavage at 30 mg/kg. VEGF antibody was injected intraperitoneally 3 times per week at 7.5 mg/kg. Recombinant human HGF, mouse HGF and human VEGF were obtained from R&D Systems (Minneapolis, MN, USA). All information of primary antibodies used for Western blot and immunoprecipitation were shown on Additional file 1: Table S1. All secondary antibodies were purchased from Jackson ImmunoResearch (Philadelphia, PA, USA).

### Patients and specimens

For prognostic analysis, frozen tumor and peritumor tissues were obtained from 109 patients who underwent hepatectomy for HCC at the authors’ institute between January 2005 and December 2006. For the evaluation of MET expression and microscopic vascular invasion(MVI) in HCC patients, formalin fixed and paraffin embedded tissue samples were collected from 122 patients who received the curative liver resection for HCC at the authors’ institute between September 2014 to December 2016. The entire area of the cut surface containing the greatest tumor dimensions and noncancerous liver tissue was submitted for the histologic examination. The clinicopathological characteristics of these patients were presented in Additional file 1: Table S2, S3. All patients were diagnosed with HCC, and none had received any preoperative cancer treatment. Clinical samples were collected from patients after obtaining informed consent in accordance with a protocol approved by the Ethics Committee of Fudan University (Shanghai, China).

To evaluate the MVI in HCC patients, the specimens were stained with hematoxylin and eosin. Microscopic vascular invasion(MVI) was defined as the presence of clusters of cancer cells floating in the portal vein, hepatic vein, or bile duct of the tumor and surrounding noncancerous tissues that were visible only on microscopy. All of the measurements were performed by two pathologists at Huashan Hospital, Fudan University (Shanghai, China) with more than 10 years of experience in hepatic pathology.

### Cell lines

Cell lines were described in the Additional file 2: Materials and Methods. The clinicopathological characteristics of patients, whose tissue samples were used to establish patient-derived HCC cell lines were presented in Additional file 1: Table S4.

### Western blot

Western blot was performed as previously described [16]. Briefly, the cells or the isolated independent tissues were lysed with RIPA Lysis Buffer (Santa Cruz Biotechnology, CA, USA) containing protease inhibitor (Roche Corp., Basal, Swiss) and phosphatase inhibitor (Roche Corp., Basal, Swiss). The proteins were separated by SDS–PAGE and transferred to polyvinylidene difluoride (PVDF) membranes. Membranes were blocked and blotted with the relevant antibodies. Antibody binding was detected by enhanced chemiluminescence reagent (Millipore Corp., MA, USA).

### Enzyme-linked immunosorbent assay (ELISA) and immunofluorescence analysis (IF)

Enzyme-Linked Immunosorbent Assay (ELISA) and immunofluorescence analysis (IF) were described in the Additional file 2.

### RNA isolation and real-time quantitative reverse-transcription PCR

RNA isolation and real-time quantitative reverse-transcription PCR were described in the Additional file 2.

### RNA interference

Short interfering RNA (siRNA) sequences specifically targeting human HIF-1α (5’-GGGUAAAGAACAAAACACA-3′) and mouse HIF-1α (5’-CCCATTCCTCATCCGTCAAAT-3′) were purchased from shanghai GenePham (Shanghai, China). Cells were transfected with either target-specific siRNA or a scramble control siRNA using Lipofectamine RNAi MAX reagent (Life Technologies, MD, USA) according to the manufacture’ instructions.

### DNA mutation analysis

DNA mutation analysis was described in the Additional file 2.

### MET copy number variation analysis

MET copy number variation analysis of 12 HCC cell lines and 16 Chinese patient-derived HCC cells were produced using the Affymetrix Genome-Wide Human SNP Array 6.0. The MET copy number variation analysis of mouse cell lines were produced using Affymetrix Mouse Diversity Genotyping Array. All raw data were processed with the PICNIC software program and are presented as the number of MET copies.

### Cell proliferation, colony-formation assays and capillary tube formation analysis

Cell proliferation, colony-formation assays and capillary tube formation analysis were described in the Additional file 2.

### Cell invasion and wound-healing assays

Cell invasion assay and wound-healing assays were performed as previously described [17]. The detail of cell invasion and wound healing assays was described in the Additional file 2.

### Immunohistochemical analysis and diagnostic scoring system

The detail of evaluation of immunohistochemical analysis and diagnostic scoring system were described in the Additional file 2.

### Evaluation of in vivo tumor growth and metastasis in mice models of HCC

The detail of evaluation of in vivo tumor growth and metastasis in mice models of HCC was described in the Additional file 2. Tumor volume (mm^3^) was calculated by the following formula: ab^2^/2 (where a and b refer to the largest and smallest dimensions collected every 3 days after treatment). Tumor growth inhibition (TGI%) [18] was calculated using {1-[(Tt/T0)/(Ct/C0)]/1-[C0/Ct]} × 100, where Tt is the tumor volume of the treated group at indicated time t; T0 is the original tumor volume of the treated animal; Ct is median tumor volume of untreated mice at time t; and C0 is the median original tumor volume of the control group.

### Statistical analysis

Statistical analyses were performed using SPSS 15.0 for Windows (SPSS, Inc., Chicago, IL). Quantitative data between groups were compared using the one-way ANOVA; Student *t* test was used to compare data between 2 groups. Categorical data were analyzed by the chi-square test or Fisher exact test. OS and cumulative recurrence rates were calculated by the Kaplan–Meier method and differences were analyzed by the log-rank test. Univariate and multivariate analyses were performed using the Cox proportional hazards regression model. A *p*-value < 0.05 was considered statistically significant.

## Results

### MET signaling activation is responsible for the increased metastatic potential induced by VEGF signaling inhibition

**Fig. 1:**
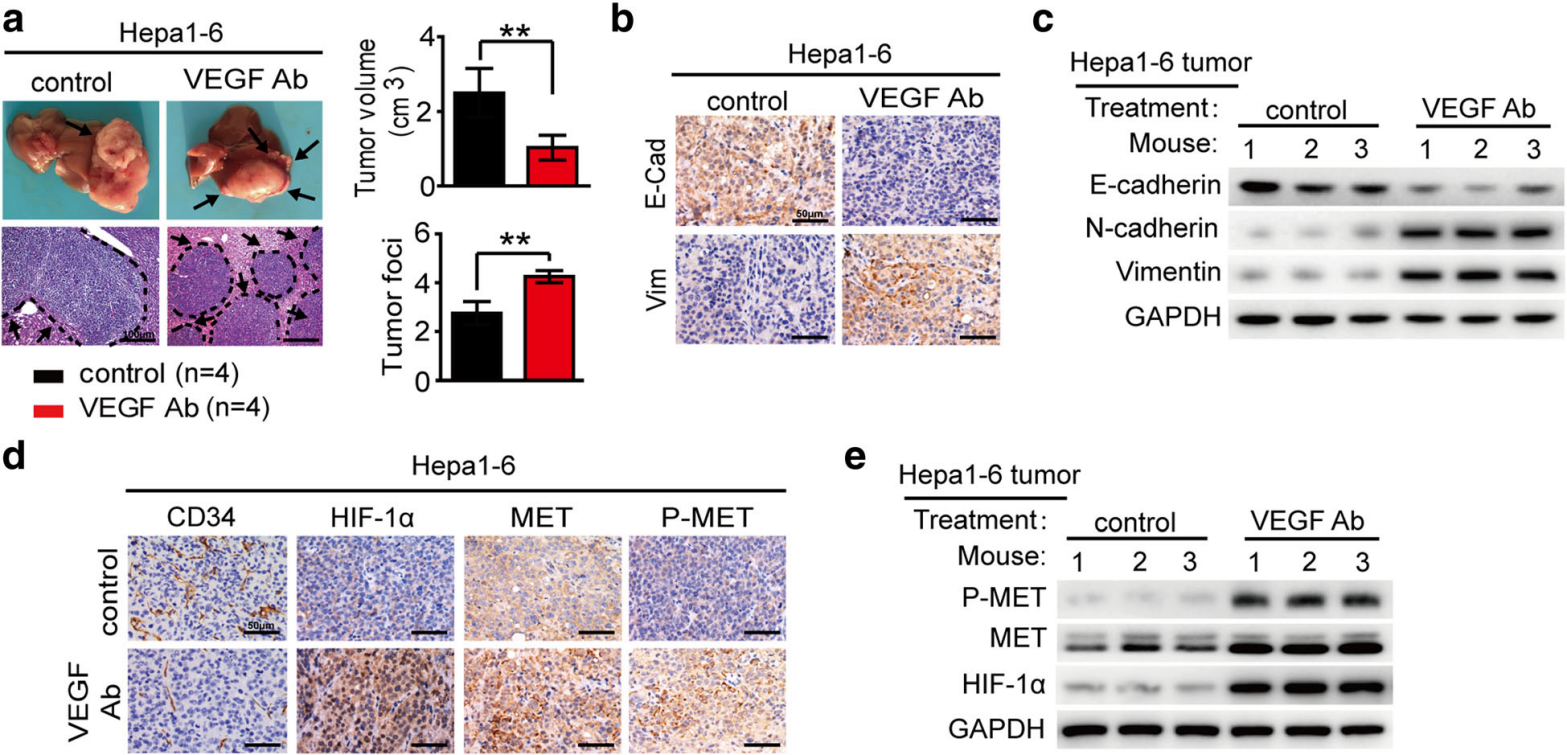
VEGF signaling inhibition increased tumor metastasis and MET activation. **a** Inhibitory effects of vehicle and VEGF antibody on HCC growth and intrahepatic metastasis in the hepa1-6 orthotopic models. Tumor volumes and numbers of tumor foci in the orthotopic implantation models were measured and quantified. **b**-**e** The effects of vehicle and VEGF antibody on EMT markers, tumor vascularity (CD34), hypoxia (HIF-1α), MET and P-MET expression detected by IHC and Western blot. Data are shown as the mean ± SD. Significant differences were determined using Student’s *t* test. *: *P* < 0.05; **: *P* < 0.01; NS: No Significance

In hepa1-6 orthotopic model, treatment with VEGF antibody significantly inhibited tumor growth. The sectional areas of tumors were 58.9% less in VEGF antibody treated group compared with control group. Though the growth was inhibited, tumors treated with VEGF antibody appeared to be more invasive as indicated by the irregularity of tumor border. The intrahepatic metastases (IHM) indicated by multiple tumor foci in liver were also significantly increased after VEGF antibody treatment (Fig. 1a). Furthermore, IHC and Western blot demonstrated that VEGF antibody treatment induced epithelial-mesenchymal transition (EMT) in tumors (Fig. 1b, c; Additional file 3: Figure S1A, B). These results indicated that VEGF antibody treatment increased the invasive and metastatic abilities of HCC cells.

Emerging evidences suggest that the promoting effect on cancer metastasis caused by angiogenesis inhibition is due to the hypoxia [19], and tumor vascular pruning resulted from the inhibition of VEGF pathway triggers upregulation of MET expression [13]. We hypothesized that hypoxia induced by VEGF antibody treatment might activate MET signaling which led to the increased potential of tumor invasion and metastasis. We used IHC and Western blot to detect the expression levels of HIF-1α, total-MET and P-MET in tumors after VEGF antibody treatment. The results showed that the positive staining for total-MET and P-MET was much stronger and more widespread in VEGF antibody-treated tumors, which was accompanied by vascular pruning (CD34 immunoreactivity) and intratumoral hypoxia (HIF-1α immunoreactivity), compared with the controls (Fig. 1d, e; Additional file 3: Figure S1C-F). We also observed a significant correlation between HIF-1α and total-MET expression levels in hepa1-6 orthotopic tumors on VEGF antibody treatment (Additional file 3: Figure S1G).

**Fig. 2:**
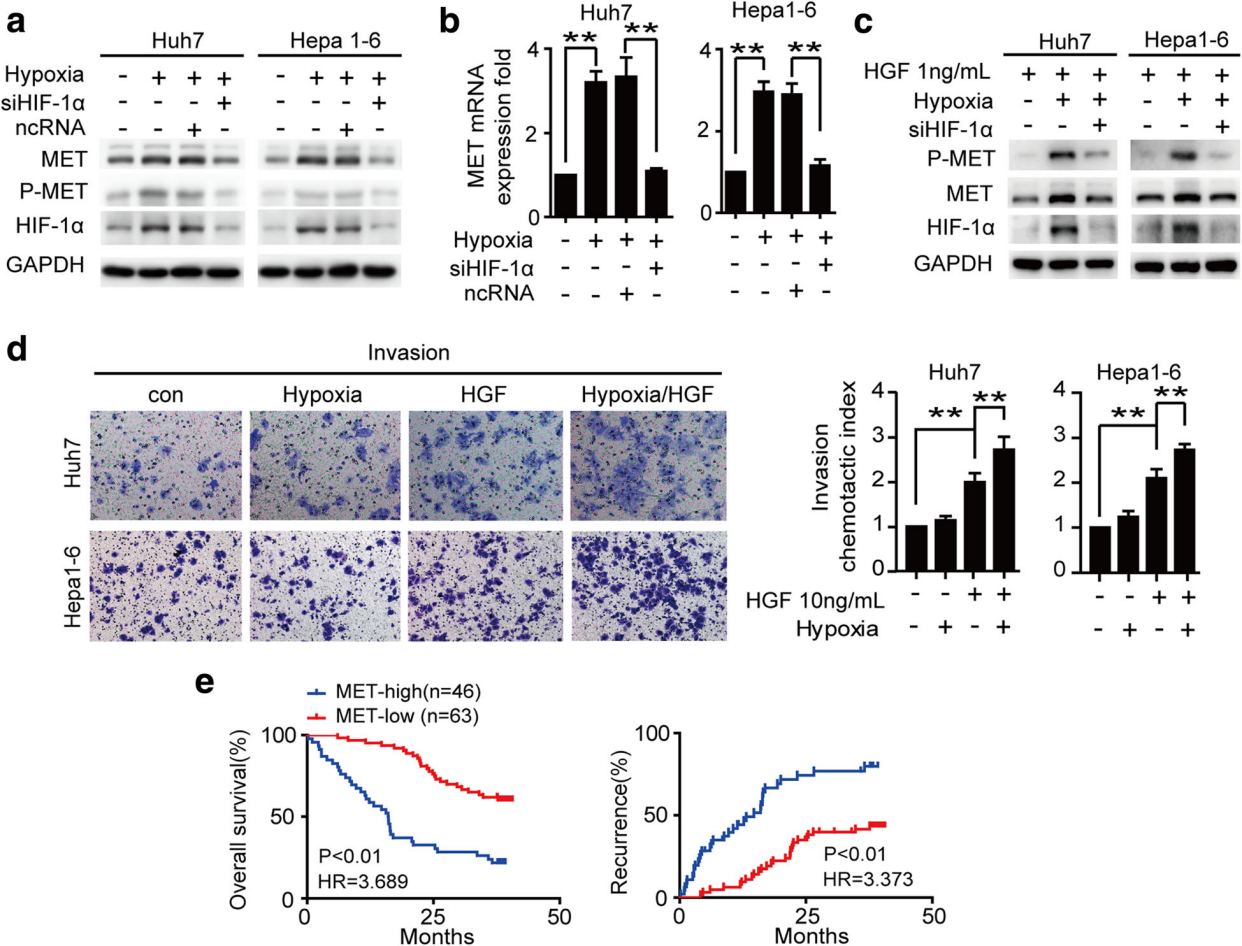
Hypoxia amplified HGF/MET signaling and augmented HCC cells invasion induced by HGF compared with normoxia. **a** Western blot detected the total-MET, P-MET and nuclear HIF-1α expression levels in HCC cells transfected with HIF-1α siRNA or NC siRNA incubated in normoxia (20% O_2_) or hypoxia (1% O_2_) for 24 h. **b** The total-MET levels in hypoxia-treated HCC cell lines transfected with HIF-1α siRNA or NC siRNA were detected by RT-PCR. **c** The total-MET, P-MET and nuclear HIF-1α protein levels were detected by Western blot in HCC cells incubated in normoxia (20% O_2_) or hypoxia (1% O_2_) conditions after stimulation with low concentration of HGF (1 ng/ml) for 24 h. **d** HCC cells seeded onto a layer of Matrigel were serum-starved and incubated in normoxia or hypoxia. After 24 h, cells were stimulated with HGF(10 ng/ml) in the same conditions for additional 24 h. The chemotactic index was calculated as the ratio of the number of cells that migrated to different amboceptor-containing wells divided by the number of cells that migrated to cultured medium alone. **e** The Kaplan–Meier survival curve of HCC patients with low (Tumor/Peritumor< 2-fold, *n* = 63) and high (Tumor/Peritumor≥2-fold, *n* = 46) transcriptional levels of MET. Data are shown as the mean ± SD. Significant differences were determined using one-way ANOVA. *: *P* < 0.05; **: *P* < 0.01; NS: No Significance

To further determine whether hypoxia had a direct effect on MET activation, we exposed HCC cell lines to hypoxic condition. We found that hypoxia induced HIF-1α nuclear accumulation and subsequently increased the total-MET level, but had no significant effect on MET tyrosine phosphorylation (Fig. 2a, b). As shown in Fig. 2c, hypoxia dramatically amplified the response of HCC cells to HGF, whereas knockdown of HIF-1α neutralized the hypoxia-induced expression of total-MET or HGF-induced MET tyrosine phosphorylation. In addition, Western blot assay revealed that HCC cells treated with extrinsic HGF (Huh7-HGF and Hepa1-6-HGF cells) showed typical EMT phenotypes i.e. downregulation of E-cadherin and upregulation of vimentin and N-Cadherin (Additional file 3: Figure S2). We further examined the effect of hypoxia on the spontaneous and chemokine-induced invasion of HCC cells using in vitro invasion assay. The results showed that hypoxia condition had negligible effect on the spontaneous invasion of Huh7 and Hepa1-6 cells, but it significantly increased the HGF-induced invasion in those cells (Fig. 2d).

To determine the clinical significance of MET overexpression, we correlated the MET mRNA expression with the prognosis of HCC patients. We observed that High-MET expression group (gene expression level of tumor tissue/adjacent peritumor tissue≥2) had significantly worse outcomes than those with Low-MET group (Fig. 2e). Univariate and multivariate analyses showed that the presence of MET was the independent prognostic indicator in HCC patients (Additional file 1: Table S5).

### MET signaling inhibition attenuates the metastasis-promoting effects induced by VEGF antibody

**Fig. 3:**
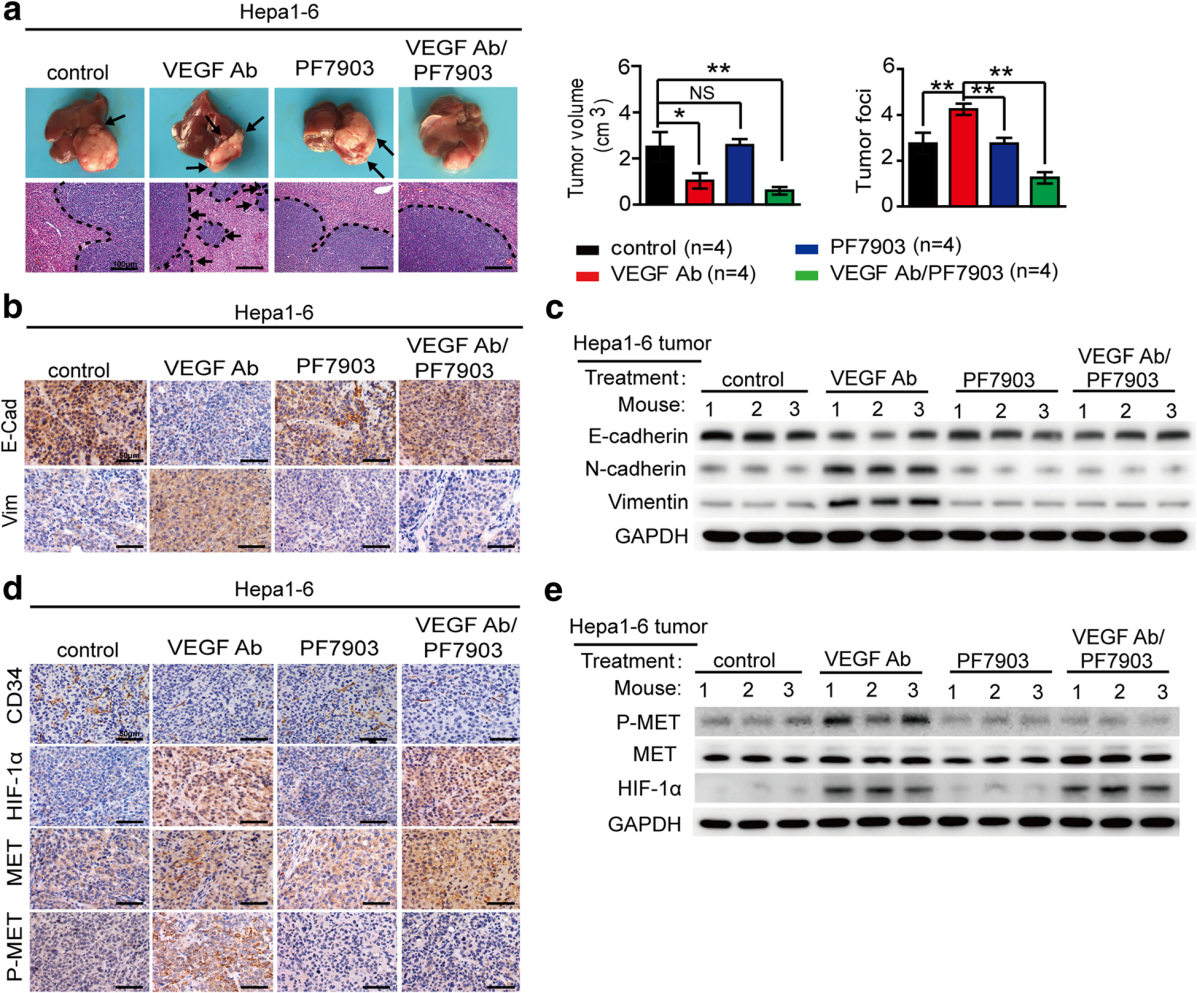
MET signaling inhibition attenuated the invasion and metastasis-promoting effects induced by VEGF inhibition in hepa1-6 orthotopic models. **a** Inhibitory effects of VEGF antibody, PF-04217903 alone, and their combination on HCC growth and intrahepatic metastasis in the hepa1-6 orthotopic models. Tumor volumes and numbers of tumor foci in the orthotopic implantation models were measured and quantified. **b**-**c** The expression levels of EMT markers in HCC tissues detected by IHC and Western blot after VEGF and/or MET signaling inhibition. **d**-**e** The effects of VEGF and/or MET inhibition on tumor vascularity (CD34), hypoxia (HIF-1α), MET and P-MET expression detected by IHC and Western blot. Data are shown as the mean ± SD. Significant differences were determined using one-way ANOVA. *: *P* < 0.05; **: *P* < 0.01; NS: No Significance

Based on the above observation, we next investigated whether concurrent inhibition of MET signaling was sufficient to attenuate the promotion of HCC invasion and metastasis induced by VEGF inhibition. We applied PF-04217903, a selective MET signaling inhibitor, in the hepa1-6 orthotopic models, and found that PF-04217903 alone did not result in obvious alterations in tumor volume and metastasis compared with the controls (Fig. 3a). However, the combined treatment with VEGF antibody and PF-04217903 resulted in significant smoother contour and fewer IHM than those treated with VEGF antibody alone (Fig. 3a; Additional file 3: Figure S3), and it also abrogated the EMT induced by VEGF antibody (Fig. 3b, c; Additional file 3: Figure S4A, B). Intriguingly, we observed that this combination significantly inhibited the P-MET expression rather than the total-MET expression levels and didn’t induce greater vascular pruning (CD34 immunoreactivity) and intratumoral hypoxia (HIF-1α immunoreactivity), compared with those treated with VEGF antibody alone (Fig. 3d, e; Additional file 3: Figure S4C-F). These findings suggested that blocking MET activation is sufficient to attenuate the enhanced metastatic potential induced by VEGF inhibition, and simultaneous blocking MET and VEGF signaling might provide more therapeutic benefit for HCC patients.

### MET and VEGFR2 dual blockade by NZ001 results in pronounced inhibition on tumor growth and metastasis of HCC

**Fig. 4:**
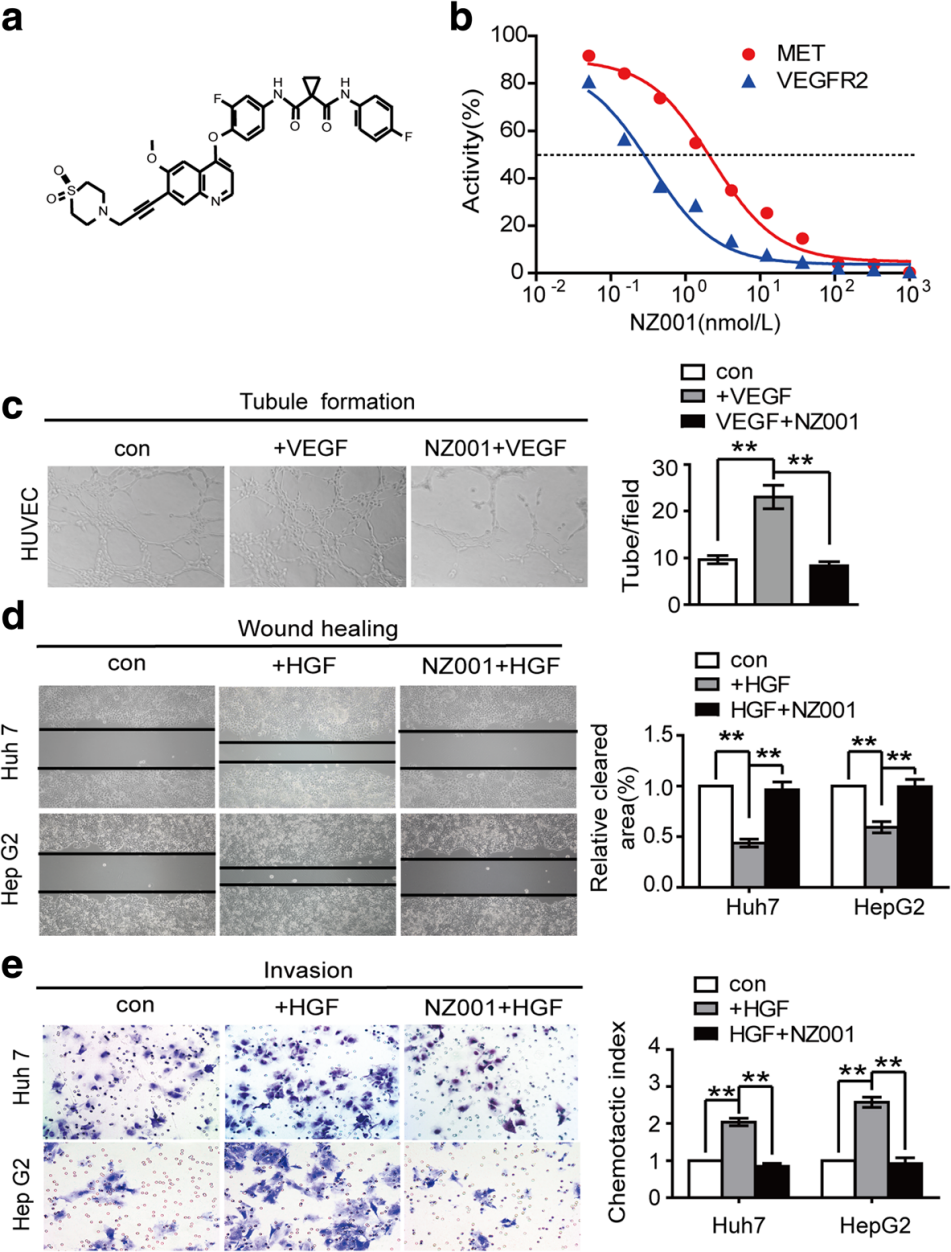
NZ001, a novel dual inhibitor of MET and VEGFR2, inhibited capillary tube formation of HUVECs and HGF-induced migration and invasion of HCC cells. **a** The molecular structure of NZ001. **b** The inhibitory effects of NZ001 on the kinase activities of MET and VEGFR2 and IC50 values determined by KinaseProfiler IC50 Express (Millipore). **c** NZ001(1umol/l) significantly inhibited VEGF(50 ng/ml)-driven capillary-like structures of HUVECs. **d** NZ001(1umol/l) significantly inhibited HGF(10 ng/ml)-stimulated migration in a wound-healing assay. **e** NZ001(1umol/l) significantly inhibited HGF(10 ng/ml)-induced cell invasion in a Transwell assay Data are shown as the mean ± SD. Significant differences were determined using one-way ANOVA. *: *P* < 0.05; **: *P* < 0.01; NS: No Significance.

Because of the favorable effects of combination therapy observed above, we further analyzed the potential action of NZ001 (Fig. 4a, b and Additional file 1: Table S6), a novel ATP-competitive multi-targeted kinase inhibitor that simultaneously blocks MET and VEGFR2, in HCC. We observed that VEGFR2 was pronounced expressed in vasculature endothelial cells, which could be phosphorylated by its ligand VEGF. However, no VEGFR2 expression was detected in cultured HCC cells (Additional file 3: Figure S5A). So, the effects of NZ001 on VEGFR2 signaling were tested in vasculature endothelial cells. In a cytokine-stimulated tyrosine kinase activity assay we found that NZ001 blocked VEGF-induced phosphorylation of VEGFR2 and its downstream effectors STAT3, ERK1/2 and AKT in HUVEC cells (Additional file 3: Figure S5B). Consistent with this, NZ001 significantly prevented the VEGF-induced formation of vessel-like structures as observed by the elongation and alignment of the cells at the indicated concentrations (Fig. 4c). We also found that NZ001 treatment markedly inhibited HGF-induced phosphorylation of MET and its resultant downstream effectors in both Huh7 and HepG2 cells in a concentration-dependent manner (Additional file 3: Figure S5C). Moreover, EMT induced by MET activation in those cells were also inhibited by NZ001 treatment (Additional file 3: Figure S6). We further tested whether NZ001 has an effect on tumor cell motility and invasion in vitro. The wound-healing assay determined that enhanced migration of HCC cells by HGF was significantly suppressed with the application of NZ001 (Fig. 4d). Furthermore, Invasion assay showed that under both normoxia and hypoxia condition, NZ001 had no demonstrable effect on spontaneous invasion of Huh7 and HepG2 cells, but significantly inhibited the HGF-induced invasion (Fig. 4e; Additional file 3: Figure S7A-B).

**Fig. 5:**
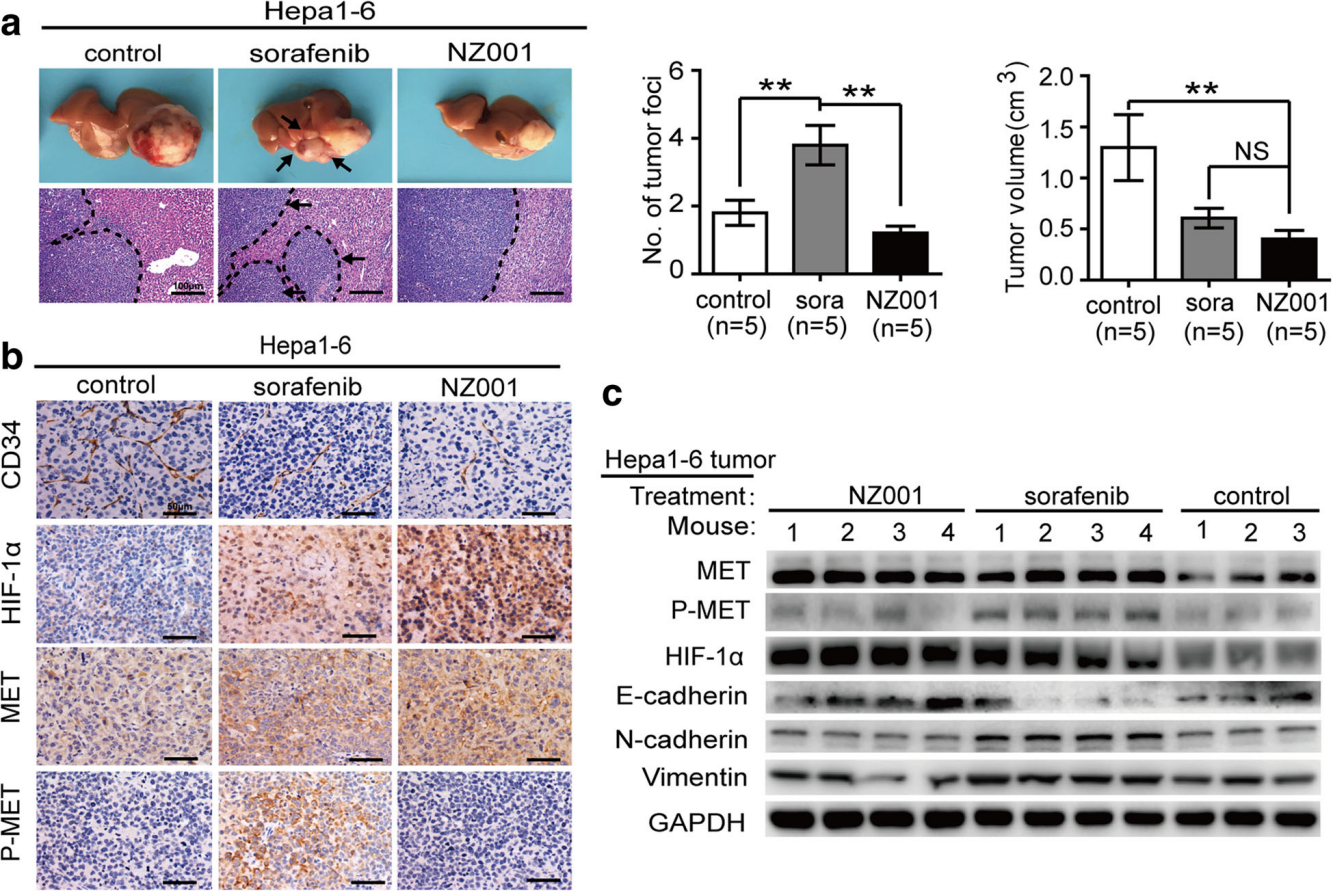
The effects of NZ001 and sorafenib on tumor growth and metastasis in orthotopic mice models of HCC. **a** Comparison of the inhibitory effects of NZ001 (30 mg/kg/d) and sorafenib (30 mg/kg/d) in the hepa1-6 orthotopic implantation models. Tumor volumes and number of tumor foci in liver were measured and quantified. **b** The effects of NZ001 and sorafenib on tumor vascularity (CD34), hypoxia (HIF-1α), total-MET and P-MET expression levels were detected by IHC. **c** Western blot detected EMT markers (E-cadherin, N-cadherin and vimentin), HIF-1α, total-MET and P-MET expression levels in hepa1-6 xenograft tumors treated with sorafenib and NZ001. Data are shown as the mean ± SD. Significant differences were determined using one-way ANOVA. *: *P* < 0.05; **: *P* < 0.01; NS: No Significance

We further observed the effects of NZ001 and sorafenib treatment on HCC growth and intrahepatic metastasis in hepa1-6 orthotopic models. Compared with vehicle controls, both the NZ001 and sorafenib treatment significantly decreased the tumor size (Fig. 5a; Additional file 3: Figure S8). Despite the smaller size, tumors treated with sorafeinb appeared to be more invasive as judged by the irregularity of tumor border, and higher intrahepatic metastases indicated by multiple tumor foci in liver (Fig. 5a; Additional file 3: Figure S8). However, besides the smaller tumor size, tumors treated with NZ001 had smoother border and didn’t enhance the intrahepatic metastases compared with vehicle controls (Fig. 5a; Additional file 3: Figure S8). In addition, both NZ001 and sorafenib treatment induced a significant decrease of MVD, increased intratumoral hypoxia and elevated total-MET levels in HCC tissues compared with the controls (Fig. 5b; Additional file 3: Figure S9A-C). However, only sorafenib, but not NZ001 treatment, increased the P-MET levels in tumor cells (Fig. 5b; Additional file 3: Figure S9D). Western blot showed a higher level of E-cadherin, decreased levels of N-cadherin and vimentin in NZ001-treated tumors compared with sorafenib-treated groups (Fig. 5c).

To further determine the effects of NZ001 on extrahepatic metastasis of HCC, MHCC-97H cells were directly injected into the tail vein of nude mice. Starting from first week, mice were treated with NZ001 for up to 3 weeks. We found that metastases in the lung and liver were reduced by 86.2% and 82.2% respectively in the NZ001 treated group (Additional file 3: Figure S10A-B). Additionally, the potential anti-metastatic function of NZ001 was confirmed by the significant difference in the whole-lung wet weights between control and NZ001 treated groups (Additional file 3: Figure S10A).

Taken together, these results indicated that NZ001, a multitargeting RTK inhibitor that blocks both MET and VEGF signaling, has more significant inhibitory effect on tumor growth without inducing invasion and metastasis frequently observed after sorafenib treatment.

### MET amplification and overexpression are associated with the sensitivity of HCC cells to MET and VEGFR2 inhibitors

**Fig. 6:**
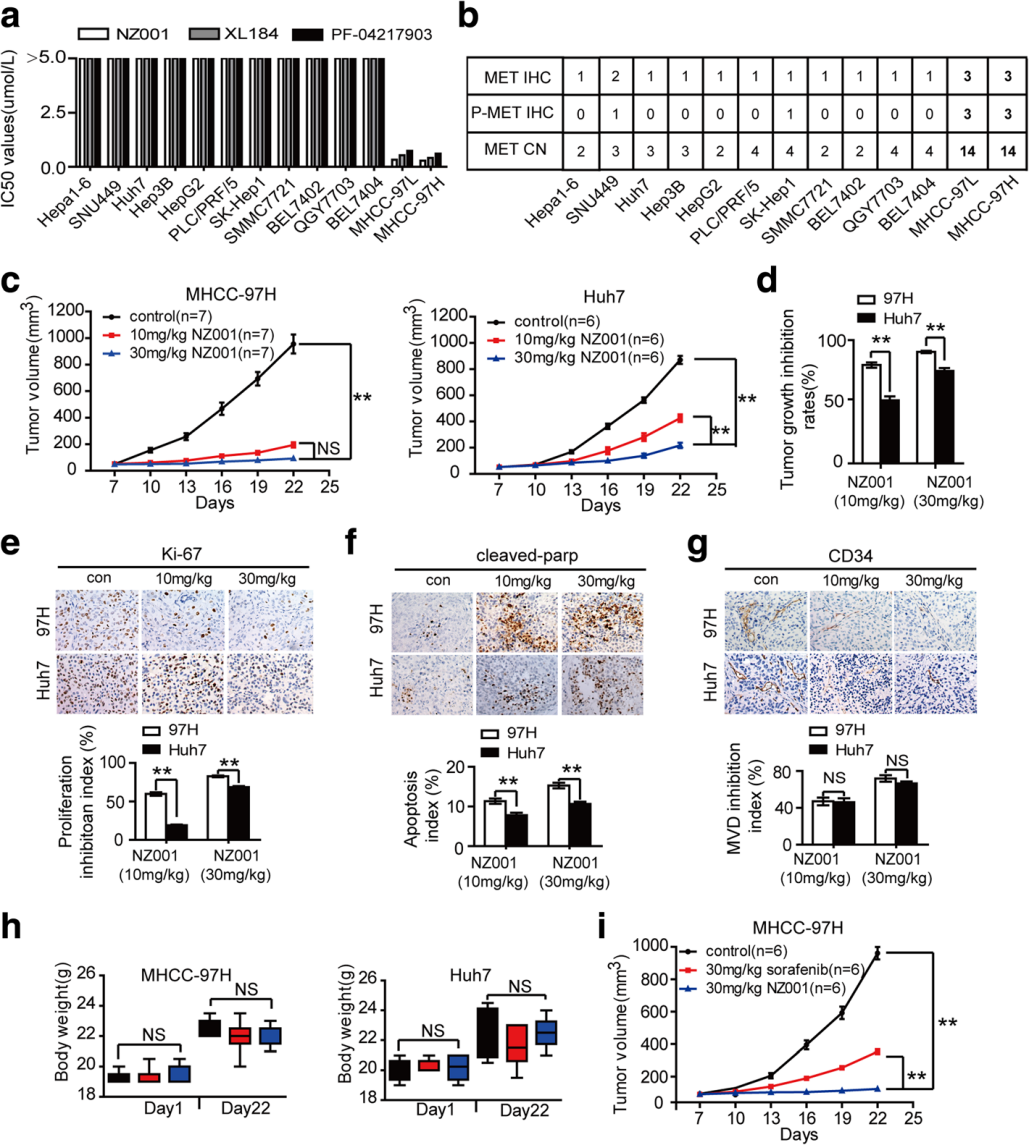
MET amplification and protein overexpression predicted the sensitivity to MET inhibitors. **a** The IC50 values of NZ001, PF-04217903 and XL184 in a panel of HCC cell lines were determined by CCK-8 assay. **b** The association of MET gene copy number in HCC cell lines and MET/P-MET protein expression levels detected by IHC in subcutaneous tumor tissues with the sensitivity to NZ001 treatment in HCC cell lines. **c** Comparison of tumor growth rates in subcutaneous implantation models of MHCC-97H and Huh7 cells after treatment with different dose of NZ001 (either 10 or 30 mg/kg daily for 14 days). Tumor volumes were measured and recorded every 3 days from initial treatment to tumor harvest. **d** Tumor growth inhibition (TGI) was measured at the end of the experiment. **e**-**g** Effects of NZ001 on proliferation, apoptosis and angiogenesis of MHCC-97H and Huh7 xenografts determined by IHC. **h** Body weights were measured and recorded from initial treatment to tumor harvest. **i** Comparison of the inhibitory effects of NZ001 and sorafenib in MHCC-97H xenografts models (30 mg/kg of NZ001 or sorafenib for 14 days). Tumor volumes were measured and recorded every 3 days from initial treatment to tumor harvest. Data are shown as the mean ± SD. Significant differences were determined using one-way ANOVA. *: *P* < 0.05; **: *P* < 0.01; NS: No Significance

To identify the biomarkers for determining sensitivity of HCC to this novel agent, we treated 13 HCC cell lines with NZ001, selective MET tyrosine kinase inhibitor PF-04217903 and another putative MET and VEGFR2 tyrosine kinase inhibitor XL-184 in an in vitro antiproliferation screen. After 48 h of treatment, the IC50 values were calculated. Compared with other cells, both MHCC-97 L and MHCC-97H cells were highly sensitive to NZ001, PF-04217903 and XL184 (Fig. 6a). NZ001 markedly inhibited the colony formation of the sensitive cell lines (MHCC-97 L and MHCC-97H), but exerted no obvious effects on insensitive cell lines (Huh7 and HepG2) (Additional file 3: Figure S11A). These results were also confirmed by alterations of cyclin D1 and cleaved-PARP detected by Western blot (Additional file 3: Figure S11B, C).

To explore the possible reason involved for various sensitivities of different HCC cells to MET inhibitors, we analyzed the exon14 mutation of *MET* (which could distinguish a unique subset of non-small cell lung carcinoma patients likely to benefit from MET inhibitors [20, 21]), copy numbers and expression levels of MET/P-MET in HCC cells [22, 23]. We didn’t observe any mutation on *MET* exon 14 in both sensitive and insensitive HCC cells by sanger sequencing (Additional file 3: Figure S12). However, MET gene copy number (CN > 4) was increased in both MHCC-97 L and MHCC-97H cell lines compared with insensitive cell lines (Fig. 6b). Furthermore, the IHC assay revealed that sensitive HCC cells showed higher levels of total MET and P-MET expression, which was defined as greater than 50% of cells with strong membrane staining (IHC 3+) in tumor xenografts. (Fig. 6b; Additional file 3: Figure S13). The ELISA assay also demonstrated that total MET and P-MET levels but not HGF, were significantly elevated in both sensitive cell lines compared with insensitive cells (Additional file 3: Figure S14A, B).

Having observed that MET and VEGFR2 inhibitors inhibited proliferation of *MET*-amplified and MET/P-MET-high–expressing HCC cells in vitro, we next evaluated the potential action of NZ001 to MHCC97H (MET amplification and high MET protein expression) and Huh7 (non-MET amplification and low MET protein expression) xenograft models in vivo. The results demonstrated that treatments with 10 and 30 mg/kg of NZ001 led to more tumor growth inhibition (TGI) of MHCC-97H xenografts than Huh7 xenografts (Fig. 6c, d; Additional file 3: Figure S15A, B). IHC analysis further revealed that NZ001 treatment resulted in more pronounced reduction of proliferation (Ki-67–positive cells) and increased apoptosis (the cleaved-PARP-positive cells) of MHCC-97H xenografts than Huh7 xenografts (Fig. 6e, f). Whereas, the MVDs of both MHCC-97H and Huh7 xenografts were significantly decreased after NZ001 treatment (Fig. 6g). In addition, NZ001 was well tolerated in mice indicated by the stable and/or increasing body weights during the treatment period (Fig. 6h). Moreover, NZ001 showed a better tumor inhibition efficacy than sorafenib in MHCC-97H xenografts (Fig. 6i**)**. These results suggested that the novel MET and VEGFR2 blockade, NZ001, significantly inhibited tumor growth and angiogenesis of HCC, especially in tumors with MET amplification and high MET protein expression.

**Fig. 7:**
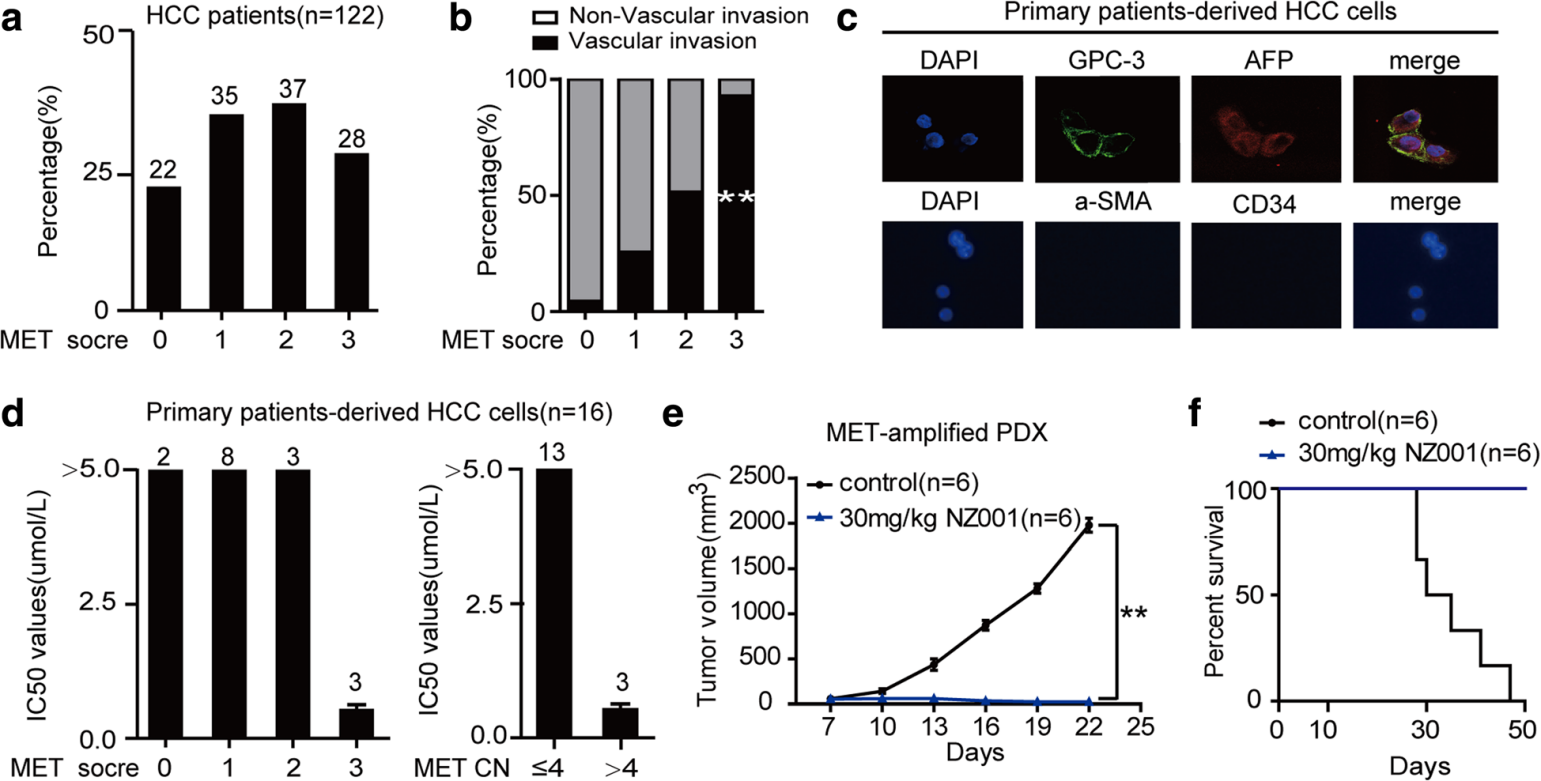
The antitumor effects of NZ001 in PDX model. **a** The MET protein expression in the 122 hepatocellular carcinoma samples were analyzed by IHC. The numbers on the top of the columes: the number of patients with different MET expression. **b** Vascular invasion rate in HCC samples from different MET expression groups. Significant differences were determined using chi-square test. **c** Immunofluorescence analysis was performed to detect the expression of HCC markers (AFP and GPC-3), fibroblast marker (a-SMA) and endothelial marker (CD34) in primary cancer cells from fresh HCC samples. **d** The effect of NZ001 on primary cancer cells from patients with HCC. The numbers on the top of the columes: the number of patients with different MET expression. **e** PDX models of HCC with *MET*-amplification and MET-overexpression were treated daily with vehicle or 30 mg/kg of NZ001 for 2 weeks. Tumor volumes were measured and recorded every 3 days from initial treatment to tumor harvest. **f** Kaplan–Meier survival curves of PDX models of HCC with *MET*-amplification and MET-overexpression treated daily with 30 mg/kg of NZ001. Data are shown as the mean ± SD. *: *P* < 0.05; **: *P* < 0.01; NS: No Significance

To explore the MET protein expression levels in HCC tumors, 122 HCC tissues were analyzed by IHC. The results showed that the prevalence of HCC patients with high MET protein expression (IHC 3+) was 22.9% (28/122) (Fig. 7a). Furthermore, patients with high MET protein expression also had more vascular-invasive tumors (Fig. 7b). To examine the effect of NZ001 on HCC, we isolated cancer cells from fresh HCC clinical samples. The purity of established HCC was characterized by immunofluorescence analysis and revealed that cultured cells in vitro showed positive staining for HCC markers including AFP and GPC-3 and negative staining for fibroblast marker a-SMA and endothelial cell marker CD34 (Fig. 7c). We then analyzed the *MET* copy number and protein expression in primary HCC cells. Three out of 16 primary HCC cells exhibited *MET* gene amplification, which were also positive for elevated MET protein expression (IHC 3+) in HCC tissues, and showed higher sensitive to NZ001 treatment compared with other cells (Fig. 7d; Additional file 1: Table S7, 8). Furthermore, PDX (patient–derived tumor xenograft) model experiment showed that NZ001 had a significant inhibitory effect on tumor growth of HCC with *MET*-amplification and MET-overexpression, and also prolonged the survival of mice (Fig. 7e, f). These suggested that *MET* amplification or high MET/P-MET expression could be used to identify the patients most likely to get the optimal benefit from NZ001 treatment.

### NZ001 selectively inhibits MET phosphorylation and their downstream effectors in HCC cells with *MET*-amplification and MET-overexpression

**Fig. 8:**
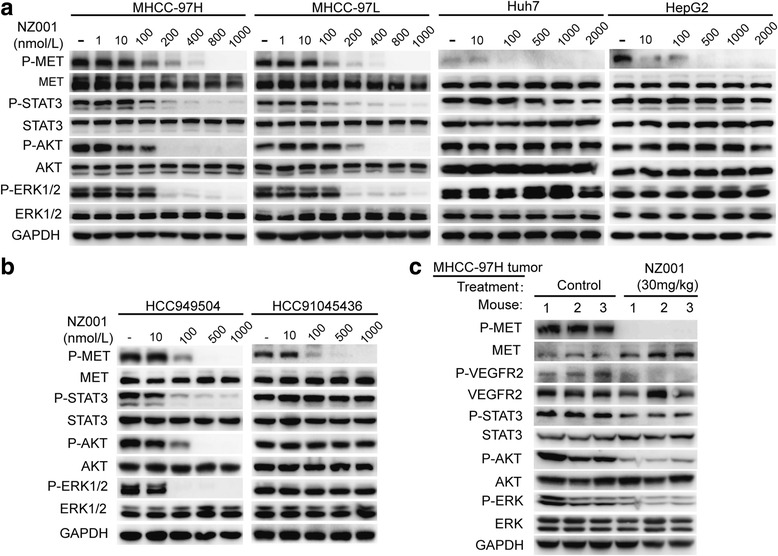
Effects of NZ001 on MET and VEGFR2 and their downstream effectors in HCC cells. **a** Western blot demonstrated that NZ001 significantly inhibited the phosphorylation of MET and its downstream effectors including STAT3, AKT and ERK1/2 in HCC cells with *MET*-amplification and MET-overexpression (MHCC-97 L, MHCC-97H) in a dose-dependent manner, rather than in those without *MET*-amplification and MET-overexpression (Huh7, HepG2). Cells were treated with the indicated concentrations of NZ001 or 0.1% DMSO in DMEM containing 10%FBS for 5 h before protein extraction. **b** The effect of NZ001 on phosphorylation of MET and downstream effectors in patient-derived HCC cells(HCC949504:CN > 4, MET/P-MET IHC 3+; HCC91045436:CN < 4, MET/P-MET IHC 2+). **c** The effect of NZ001 on phosphorylation of VEGFR2 and MET phosphorylation and their downstream effectors in tumors from the established MHCC-97H xenografts (*n* = 3/group) treated daily with an oral dose of NZ001 at 30 mg/kg or vehicle for 3 days

Next, we investigated the effect of NZ001 treatment on MET activity and its downstream signal pathways. We found that NZ001 potently inhibited P-MET in all HCC cell lines with detectable levels of P- MET. However, dose-dependent inhibition of P-STAT3, P-AKT, and P-ERK was only found in those with *MET* amplification and high MET/P-MET protein levels (Fig. 8a). Consistent to these findings, inhibition of MET and its downstream signaling by NZ001 was also only observed in the patients-derived HCC cells with *MET*-amplified and high MET/P-MET expression (Fig. 8b), pointing close association between inhibition of MET downstream signaling by NZ001 and *MET*-amplification and MET/P-MET high expression.

To further examine if NZ001 inhibits VEGFR2 and MET signaling activity in vivo, established xenografts of MHCC-97H with *MET*-amplified and MET/P-MET overexpression were treated with an oral dose of vehicle or NZ001 at 30 mg/kg for 3 days. As shown in Fig. 8c, P-VEGFR2, P-MET and their downstream effectors, such as P-STAT3, P-AKT and P-EKR signaling were dramatically inhibited after NZ001 treatment in NZ001-sensitive MHCC-97H xenografts. Moreover, IHC assay also showed a dose-dependent reduction of P-MET positive cells after NZ001 treatment (Additional file 3: Figure S16).

## Discussion

Suppressing neo-angiogenesis has become an important cancer therapeutic approach. However, accumulating preclinical and clinical evidences indicate that there are several limitations with this approach, including the limited survival benefit, lack of specific biomarker for identifying the patients likely to be advantageous, primary or acquired resistance, and inducing more invasive or metastatic behavior of cancers [19]. So, it is urgent to explore the involved mechanisms and develop novel strategies to overcome the side effects of anti-angiogenic therapy such as cancer invasion and metastasis.

Agents that block the actions of VEGF not only cause vascular pruning but also result in hypoxia [24]. Intratumoral hypoxia, which is known as a major stimulator of VEGF and consequent angiogenesis, is associated with a greater risk of metastasis and less favorable prognosis [25]. One of crucial mechanisms is that hypoxia increases MET expression in tumor cells through HIF-1α binding to the MET promoter and induces transcriptional activation of MET, which drives cell motility and invasion [26]. These open issues led us to study about whether MET activation under hypoxia inducing a tumor-invasive switch in HCC is a mechanism of refractory to antiangiogenic treatment and whether this form of evasive resistance can be prevented or reversed by inhibition of MET in HCC. Our in vivo experiment demonstrated that application of VEGF signaling monoclonal antibody slowed tumor growth but promoted invasion of tumor cells into the adjacent normal tissue and increased the number of intrahepatic metastases. We illustrated that intratumoral vascular pruning induced by VEGF Ab resulted in hypoxia, HIF-1α nuclear accumulation, MET activation and conversion to a more mesenchymal tumor cell phenotype. In vitro assays demonstrated that hypoxia caused by VEGF signaling inhibition induced HIF-1α nuclear accumulation leading to elevated total-MET expression, which synergized with HGF to enhance the invasion of HCC cells. We also observed that patients with MET overexpression had more vascular-invasive tumors and shorter survival. Importantly, the exaggerated aggressiveness of tumors was circumvented when the selective MET inhibitor, PF-04217903, was co-administered with the VEGF Ab. PF-04217903 given in combination with VEGF antibody also attenuated the MET phosphorylation and the epithelial to mesenchymal transition. Taking together, these findings indicated that VEGF inhibition resulted in hypoxia and activated MET signaling enhancing the metastatic potential of HCC, and MET signaling inhibition attenuated the metastasis-promoting effects of HCC induced by VEGF inhibition.

Because of these favorable effects of the combination treatment, we designed a chemical compound, NZ001, which could simultaneously block both MET and VEGFR2 signaling. The structure of NZ001 had less similarity to existed VEGFR or MET selective inhibitors and the manner of kinase inhibition by NZ001 was shown to be ATP antagonism with the IC50 values against MET and VEGFR2 in the low nanomolar or subnanomolar range. We examined the effect of NZ001 on the spontaneous and cytokine-induced invasion in HCC cell lines under normoxia and hypoxia condition. Our results showed that NZ001 had minimal impact on the spontaneous invasion of Huh7 and HepG2 cells but strongly reduced HGF-stimulated invasion in those cells under both normoxia and hypoxia condition, which was accompanied by a marked inhibition of HGF-stimulated phosphorylation of MET and its downstream effectors STAT3, AKT and ERK. However, in non-cytokine stimulated Huh7 and HepG2 cells, NZ001 had no demonstrable effect on the phosphorylation of STAT3, AKT or ERK, which indicated that the invasion ability of those cells was depended on the downstream signaling of MET. In hepa1-6 orthotopic mice model, we found that inhibition of VEGF signaling by multitargeted RTK inhibitor sorafenib slowed tumor growth but promoted invasion of tumor cells into the adjacent normal tissue and increased the number of intrahepatic metastases. These effect also accompanied with vascular pruning, hypoxia, HIF-1a accumulation, MET activation and conversion to a more mesenchymal tumor cell phenotype. However, NZ001 caused greater inhibition of hepa1-6 tumor growth and invasion than control- and sorafenib-treatment. Administration of NZ001 blocked MET activation and epithelial to mesenchymal transition caused by VEGF antibody and sorafenib. In experimental metastases model, mice treated with NZ001 showed fewer HCC metastatic foci in lung and liver tissues compared with control treated groups. These suggested that NZ001 profoundly inhibited the tumor growth and metastasis of HCC indicating advantages over sorafenib.

Despite significant preclinical data supporting the role of MET as a potential oncogenic driver in HCC, the clinical data obtained with application of MET inhibitors in HCC was not appreciable [27, 28]. The hidden reasons behind this still unclear, but consensus exists on distinguishing patients who could get the optimal benefit from MET targeted therapy. To identify the predictive biomarkers for MET targeted therapy in HCC, we employed SNP 6.0 assay, sanger sequencing and IHC. We then characterized the MET gene copy numbers, mutation status and quantified HGF/MET/P-MET protein levels in a panel of HCC cell lines and HCC patient-derived cells. We found that HCC cells with *MET* amplification and MET/P-MET overexpression exhibited higher sensitivity to MET inhibitors in vitro. However, those with mid-level of MET/P-MET expression (IHC 2+), which have been used as selection criteria for MET targeted therapy in clinical trials of lung carcinoma [29], showed poor response to MET inhibition. Notably, *MET* exon 14 alterations, which resulted in increased MET protein levels due to disrupted ubiquitin mediated degradation and considered as a viable therapeutic target in NSCLC [20, 21], was not detected in those sensitive HCC cell lines. Furthermore, though elevated circulating HGF levels were observed in patients with HCC [30, 31], the levels of HGF were also not significantly different in sensitive and non-sensitive HCC cell lines. Consistent to in vitro study, NZ001 treatment to MET amplified MHCC-97H xenografts resulted more pronounced TGI compared with non-MET amplified Huh7 xenografts. Both of MHCC-97H and Huh7 xenografts treated with NZ001 showed no significant difference in vessel pruning, but it exerted higher apoptosis and suppression of cell cycle on MHCC-97H xenografts than on Huh7 xenografts. Based on our findings, we proposed that the antitumor effect of NZ001 on MET amplified MHCC-97H xenografts was likely to be mediated by inhibiting tumor angiogenesis through VEGFR2 inhibition (anti-angiogenesis effect) and by directly inhibiting tumor cell proliferation (anti-proliferation effect). For non-MET amplified HCC such as Huh7 tumors, impeding stromal angiogenesis through VEGFR2 inhibition (anti-angiogenesis effect) contributed to the dominant abrogation of tumor growth. Importantly, NZ001 also showed more profound TGI than sorafenib in MET-amplified MHCC-97H xenografts. These findings suggested that MET amplification and overexpression, rather than MET mutation and HGF expression, could be used to identify the subgroup of HCC patients most likely to get the optimal benefit from NZ001 treatment.

Moreover, both in vitro and in vivo assays demonstrated that NZ001 selectively inhibited MET and VEGFR2, and their downstream effectors, such as P-STAT3, P-AKT and P-ERK, in a dose-dependent manner only to HCCs with *MET* amplification and MET/P-MET overexpression. These further supported interrelation between NZ001 sensitivity and *MET*-amplification/MET overexpression and verified NZ001 as a simultaneous inhibitor of both MET and VEGFR2 signaling.

## Conclusions

In summary, we demonstrated that the activation of MET was responsible for the metastasis-promoting effects induced by VEGF inhibition, and MET signaling inhibition attenuated this unfavorable outcome induced by VEGF inhibition. MET and VEGFR2 dual blockade, NZ001, resulted more pronounced inhibition on tumor growth and metastasis of HCC and showed advantages over sorafenib, especially not inducing more invasive and metastatic behaviors. MET amplification and overexpression could be used to determine the subgroup of patients to obtain the most favorable outcome of NZ001 treatment.

## Additional files

Additional file 1: **Table S1.** Primary Antibodies for WB, IF and IHC. **Table S2.** Clinicopathological characteristics of HCC patients for analyzing the clinical significance of MET mRNA expression (*n* = 109). **Table S3.** Clinicopathological characteristics of HCC patients for detecting MET protein expression by IHC staining(*n* = 122). **Table S4.** Clinicopathological characteristics of HCC Patients for isolating primary HCC cells (*n* = 16). **Table S5.** Univariate and multivariate analyses of factors associated with OS and TTR. **Table S6.** Pharmacokinetic characteristics of NZ001 in mouse. **Table S7.** The analysis of correlation between MET gene amplification and MET protein expression in the 16 HCC samples. **Table S8.** The analysis of correlation between MET gene amplification and P-MET protein expression in the 16 HCC samples. (DOCX 39 kb)

Additional file 2: Supplementary Materials and Methods. (DOCX 30 kb)

Additional file 3: **Figure S1.** The E-cadherin, vimentin, CD34, HIF-1α, P-MET and total-MET expression levels in hepa1–6 orthotopic tumors after treatment with vehicle and VEGF antibody. **Figure S2.** The expression levels of E-cadherin, N-cadherin and vimentin were detected by Western blot in HCC cells starved overnight and treated with HGF (10 ng/ml) for 24 h. **Figure S3.** After 2-week treatment with VEGF antibody, PF-04217903 alone, and their combination, the mice were killed and the liver tissues were obtained. **Figure S4.** The E-cadherin, vimentin, CD34, HIF-1α, total-MET and P-MET expression levels in hepa1–6 orthotopic tumors after treatment with VEGF antibody and PF-04217903 alone, or their combination. **Figure S5.** The effect of NZ001 on VEGFR2 and MET signaling in HUVECs and HCC cells. **Figure S6.** NZ001 suppressed the HGF-induced EMT in HCC cells. **Figure S7.** Effects of NZ001 on the spontaneous and chemokine-induced invasion of HCC cell under normoxia and hypoxia condition. **Figure S8.** After 2-week treatment with sorafenib and NZ001, the mice were killed and the liver tissues were obtained. **Figure S9.** The total-MET, HIF-1α, CD34 and P-MET expression levels in hepa1–6 orthotopic tumors after treatment with VEGF antibody and PF-04217903 alone, or their combination. **Figure S10.** Effects of NZ001 on the metastasis of MHCC97H in nude mice. **Figure S11.** Effects of NZ001 on colony-formation of HCC cells. **Figure S12.** The association of *MET* exon 14 mutation determined by sanger sequencing with NZ001 sensitivity of 12 HCC cell lines. **Figure S13.** The score of MET and P-MET definition for IHC staining. **Figure S14.** The MET/P-MET/HGF expression in HCC cell lines. **Figure S15.** After 2-week treatment with different concentrations of NZ001, the mice were sacrificed and the tumors were obtained. **Figure S16.** IHC assay showed that MET phosphorylation was greatly inhibited after NZ001 treatment in MHCC-97H xenograft tumors. (DOCX 5327 kb)

## Abbreviations

ELISA: Enzyme-linked immunosorbent assay
HCC: Hepatocellular carcinoma
HGF: Hepatocyte growth factor
HR: Hazard ratio
HUVECs: Human vascular endothelial cells
IHC: Immunohistochemistry
MET: Mesenchymal-epithelial transition factor
SD: Standard deviation
VEGFR2: Vascular endothelial growth factor receptor 2
