# Supplementary material for: The dual blockade of MET and VEGFR2 signaling demonstrates pronounced inhibition on tumor growth and metastasis of hepatocellular carcinoma
Source: J Exp Clin Cancer Res. 2018 Apr 30;37:93. doi: 10.1186/s13046-018-0750-2 (PMC5925844; doi:10.1186/s13046-018-0750-2)
Supplement: Supplementary file 1 — Table S1. Primary Antibodies for WB, IF and IHC. Table S2. Clinicopathological characteristics of HCC patients for analyzing the clinical significance of MET mRNA expression (n = 109). Table S3. Clinicopathological characteristics of HCC patients for detecting MET protein expression by IHC staining(n = 122). Table S4. Clinicopathological characteristics of HCC Patients for isolating primary HCC cells (n = 16). Table S5. Univariate and multivariate analyses of factors associated with OS and TTR. Table S6. Pharmacokinetic characteristics of NZ001 in mouse. Table S7. The analysis of correlation between MET gene amplification and MET protein expression in the 16 HCC samples. Table S8. The analysis of correlation between MET gene amplification and P-MET protein expression in the 16 HCC samples. (DOCX 39 kb) [file 13046_2018_750_MOESM1_ESM.docx]

**Additional file 1**

**Table S1.** Primary Antibodies for WB, IF and IHC

| Protein | Concentration for WB | Concentration for IHC | Concentraion for IF | specificity | Company |
| --- | --- | --- | --- | --- | --- |
| MET | 1:1000 | 1:200 | / | Mouse; Rabbit | SantaCruz(SC-8057);CST(8198) |
| P-MET（Tyr1234/1235） | 1:1000 | 1:200 | / | Rabbit | CST(3077) |
| VEGFR2 | 1:1000 | / | / | Rabbit | CST(9698) |
| P-VEGFR2 | 1:1000 | / | / | Rabbit | CST(2478) |
| CD34 | / | 1:200 | / | Rabbit | Abcam(EP373Y) |
| Ki-67 | / | 1:200 | / | Rabbit | CST(9027) |
| AKT | 1:1000 | / | / | Rabbit | CST(4685) |
| P-AKT  (ser473) | 1:1000 | / | / | Rabbit | CST(4060) |
| STAT3 | 1:1000 | / | / | Mouse | CST(9139) |
| P-STAT3  (Tyr705) | 1:1000 | / | / | Rabbit | CST(9145) |
| ERK | 1:1000 | / | / | Rabbit | CST(4695) |
| P-ERK（Thr202/Thr204） | 1:1000 | / | / | Rabbit | CST(4370) |
| E-Cadherin | 1:1000 | 1:200 | / | Rabbit | CST(3195) |
| N-cadherin | 1:1000 | / | / | Rabbit | Abcam(ab18203) |
| Vimentin | 1:1000 | 1:200 | / | Rabbit | CST(5741) |
| HIF-1α | 1:500 | 1:200 | / | Mouse | Abcam(ab113642) |
| GAPDH | 1:3000 | / | / | Rabbit | Proteintech（10494-1-AP） |
| AFP | / | / | 1:500 | Mouse | Abcam(ab3980) |
| GPC-3 | / | / | 1:500 | Rabbit | Abcam(ab207080) |
| a-SMA | / | / | 1:500 | Rabbit | CST(19245) |
| CD34 | / | / | 1:500 | Mouse | Abcam(ab54208) |

**Table S2** Clinicopathological characteristics of HCC patients for analyzing the clinical significance of MET mRNA expression (n=109)

| Characteristics | Case number | percentage |
| --- | --- | --- |
| Sex |  |  |
| Female | 13 | 11.9 |
| Male | 96 | 88.1 |
| Age(years) |  |  |
| <50 | 44 | 40.4 |
| ≥50 | 65 | 59.6 |
| HBsAg |  |  |
| Negative | 13 | 11.9 |
| Positive | 96 | 88.1 |
| Cirrhosis |  |  |
| No | 56 | 51.4 |
| Yes | 53 | 48.6 |
| Tumor size(cm) |  |  |
| <5 | 43 | 39.4 |
| ≥5 | 66 | 60.6 |
| Tumor number |  |  |
| Single | 96 | 88.1 |
| Multiple | 13 | 11.9 |
| Microvascular invasion |  |  |
| No | 65 | 59.6 |
| Yes | 44 | 40.3 |
| Tumor differentiation |  |  |
| I/II | 41 | 37.6 |
| III/IV | 68 | 62.4 |
| TNM stage |  |  |
| I/II | 79 | 72.5 |
| III/IV | 30 | 27.5 |

HBsAg, hepatitis surface antigen;TNM,tumor-node-metastasis;

**Table S3** Clinicopathological characteristics of HCC patients for detecting MET protein expression by IHC staining(n=122)

| Characteristics | Case number | percentage |
| --- | --- | --- |
| Sex |  |  |
| Female | 24 | 19.6 |
| Male | 98 | 80.4 |
| Age(years) |  |  |
| <50 | 30 | 24.5 |
| ≥50 | 92 | 75.5 |
| HBsAg |  |  |
| Negative | 22 | 18.0 |
| Positive | 100 | 82.0 |
| Cirrhosis |  |  |
| No | 51 | 41.8 |
| Yes | 71 | 58.2 |
| Tumor size(cm) |  |  |
| <5 | 54 | 44.3 |
| ≥5 | 68 | 55.7 |
| Tumor number |  |  |
| Single | 100 | 81.9 |
| Multiple | 22 | 18.1 |
| Microvascular invasion |  |  |
| No | 67 | 54.9 |
| Yes | 55 | 45.1 |
| Tumor differentiation |  |  |
| I/II | 82 | 67.2 |
| III/IV | 40 | 32.8 |
| TNM stage |  |  |
| I/II | 86 | 70.5 |
| III/IV | 36 | 29.5 |

HBsAg, hepatitis surface antigen; TNM,tumor-node-metastasis;

**Table S4** Clinicopathological characteristics of HCC Patients for isolating primary HCC cells (n=16)

| Characteristics | Case number | percentage |
| --- | --- | --- |
| Sex |  |  |
| Female | 4 | 25 |
| Male | 12 | 75 |
| Age(years) |  |  |
| <50 | 4 | 25 |
| ≥50 | 12 | 75 |
| HBsAg |  |  |
| Negative | 5 | 31.25 |
| Positive | 11 | 68.75 |
| Cirrhosis |  |  |
| No | 3 | 18.75 |
| Yes | 13 | 81.25 |
| Tumor size(cm) |  |  |
| <5 | 2 | 12.5 |
| ≥5 | 14 | 87.5 |
| Tumor number |  |  |
| Single | 11 | 68.75 |
| Multiple | 5 | 51.25 |
| Microvascular invasion |  |  |
| No | 8 | 50 |
| Yes | 8 | 50 |
| Tumor differentiation |  |  |
| I/II | 10 | 62.5 |
| III/IV | 6 | 37.5 |
| TNM stage |  |  |
| I/II | 6 | 37.5 |
| III/IV | 10 | 62.5 |

HBsAg, hepatitis surface antigen; TNM,tumor-node-metastasis;

**Table S5** Univariate and multivariate analyses of factors associated with OS and TTR

| Variable | OS | | Time to Recurrence | |
| --- | --- | --- | --- | --- |
|  | HR(95% CI) | P | HR(95% CI) | P |
| Univariate analysis |  |  |  |  |
| Age, y, ≤50 vs >50 | 0.965(0.579-1.608) | 0.892 | 1.034(0.623-1.718) | 0.897 |
| Sex, female vs male | 1.395(0.600-3.243) | 0.439 | 1.409(0.606-3.273) | 0.426 |
| HBsAg, negative vs positive | 0.909(0.432-1.912) | 0.801 | 0.828(0.407-1.682) | 0.601 |
| Cirrhosis, no vs ye | 1.311(0.793-2.168) | 0.291 | 1.232(0.745-2.037) | 0.416 |
| Tumor size ,cm, ≤5 vs >5 | 1.871(1.078-3.246) | **0.026** | 2.240(1.276-3.931) | **0.005** |
| Tumor number, single vs multiple | 0.634(0.273-1.473) | 0.289 | 0.530(0.212-1.325) | 0.174 |
| Microvascular invasion, no vs yes | 2.295(1.384-3.806) | **0.001** | 2.255(1.361-3.737) | **0.002** |
| Tumor differentiation, I+II vs III+IV | 2.306(1.393-3.817) | **0.001** | 1.721(1.031-2.875) | **0.038** |
| TNM stage，I+II vs III+IV | 0.600(0.325-1.108) | 0.103 | 0.601(0.325-1.111) | 0.104 |
| MET, low vs high | 3.689(2.201-6.184) | **0.000** | 3.373(2.018-5.638) | **0.000** |
| Multivariate analysis |  |  |  |  |
| Tumor size ,cm, ≤5 vs >5 | 1.244(0.679-2.280) | 0.480 | 1.658(0.893-3.081) | 0.109 |
| Microvascular invasion, no vs yes | 1.746(1.007-3.026) | **0.047** | 1.961(1.133-3.393) | **0.016** |
| Tumor differentiation, I+II vs III+IV | 1.847(1.081-3.157) | **0.025** | 1.427(0.838-2.431) | 0.191 |
| MET, low vs high | 3.476(2.068-5.843) | **0.000** | 3.604(2.144-6.058) | **0.000** |

HBsAg, hepatitis surface antigen; TNM,tumor-node-metastasis;

The Kaplan–Meier curves of time-to-early tumor recurrence after HCC resection for the transcriptional levels of MET in human HCC tissues (n=109). After normalization with the corresponding peritumor (PT) tissue sample, the tumor (T) tissues that expressed low levels (T/PT < 2-fold) of MET (n=63) were compared with tumor tissues that expressed high levels (T/PT ≥2-fold) of MET (n= 46).Analysis was conducted using univariate analysis or multivariate Cox proportional hazards regression.

**Table S6** Pharmacokinetic characteristics of NZ001 in mouse

|  | Cmax | T1/2 | CL | Vz | AUC0-t | AUC0-inf | F |
| --- | --- | --- | --- | --- | --- | --- | --- |
|  | (ng/mL) | (hour) | (L/h/Kg) | (L/Kg) | (ng•hr/mL) | (ng•hr/mL) | % |
| IV 1mg/Kg | 1777 | 13.4 | 0.12 | 0.69 | 8158 | 8407 | 100 |
| PO 5mg/Kg | 2167 | 17.2 | 0.23 | 1.71 | 20623 | 21774 | 51.8 |

**Table S7** The analysis of correlation between MET gene amplification and MET protein expression in the 16 HCC samples

|  | MET classification | | | |
| --- | --- | --- | --- | --- |
| MET IHC evaluation | Normal | Gene amplification (CN>4) | Gene mutation | NZ001 efficacy |
| 0, n (%) | 2(15.4) | 0 | 0 | NO |
| 1, n (%) | 8(61.5) | 0 | 0 | NO |
| 2, n (%) | 3(23.1) | 0 | 0 | NO |
| 3, n (%) | 0(0) | 3(100) | 0 | YES |
| Total, n(%) | 13(100) | 3(100) | 0(0) |  |

**Table S8** The analysis of correlation between MET gene amplification and P-MET

protein expression in the 16 HCC samples

|  | MET classification | | | |
| --- | --- | --- | --- | --- |
| p-MET IHC evaluation | Normal | Gene amplification (CN>4) | Gene mutation | NZ001 efficacy |
| 0, n (%) | 6 (46.2) | 0 | 0 | NO |
| 1, n (%) | 5 (38.5) | 0 | 0 | NO |
| 2, n (%) | 2 (15.3) | 0 | 0 | NO |
| 3, n (%) | 0 (0) | 3(100) | 0 | YES |
| Total, n (%) | 13(100) | 3(100) | 0(0) |  |
