# Supplementary material for: The dual blockade of MET and VEGFR2 signaling demonstrates pronounced inhibition on tumor growth and metastasis of hepatocellular carcinoma
Source: J Exp Clin Cancer Res. 2018 Apr 30;37:93. doi: 10.1186/s13046-018-0750-2 (PMC5925844; doi:10.1186/s13046-018-0750-2)
Supplement: Supplementary file 2 — Supplementary Materials and Methods. (DOCX 30 kb) [file 13046_2018_750_MOESM2_ESM.docx]

**Additional file 2 Materials and Methods**

**Cell lines**

The human HCC cell lines MHCC-97L and MHCC-97H were provided by Liver Cancer Institute, Zhongshan Hospital, Fudan University (Shanghai, China). The other cell lines were purchased from the cell bank of the Chinese Academy of Sciences (Shanghai, China). These cell lines were routinely maintained in Dulbecco’s modified Eagle’s medium (DMEM) (Gibco BRL, MD, USA) supplemented with 10% (v/v) fetal bovine serum (FBS) (Gibco BRL, MD, USA) at 37°C in a humidified incubator containing 5% CO_2_. HUVECs were cultured in serum free medium for endothelial cells (Invitrogen, CA, USA), supplemented with 20% FBS, 0.1 mg/mL of heparin and 0.03 mg/mL of endothelial cell growth supplement (Upstate Biotechnology, NY, USA).

Fresh HCC tissues were obtained from the HCC patients with ‘informed consent’ in authors’ institute and used to establish patient-derived HCC cell lines according to the standard procedures. The clinicopathological characteristics of these patients are presented in Supplementary Table 4. Briefly, Fresh HCC tissues were pretreated with collagenase and cultivated on to the Matrigel coated dish for 4 to 6 passages to select the HCC cell lines. Homogenous HCC cell populations were obtained and the sustained proliferation ability (over 20 passages). The characteristics of newly established HCC cell lines were validated by detecting HCC makers such as Glypican 3 (GPC3) (Santa Cruz Biotechnology, CA, USA) and AFP (Cell Signaling Technology, MA, USA).

**Enzyme-linked immunosorbent assay (ELISA) and immunofluorescence analysis(IF)**

For enzyme-linked immunosorbent assay (ELISA), HCC cells were cultured with serum-free medium for 24 hours. The supernatants were collected and HCC cells were lysed with RIPA Lysis Buffer (Santa Cruz Biotechnology, CA, USA) containing protease inhibitors (Roche Corp., Basal, Swiss) and phosphatase inhibitors (Roche Corp., Basal, Swiss). The supernatant and the lysates were centrifuged at 12,000 rpm for 15 minutes at 4°C to remove cell debris. The levels of total-MET and phospho-MET in the cell protein extracts and HGF in the supernatant were determined using corresponding ELISA kits according to the manufacturer's instructions (total-MET ELISA, R&D Systems DYC358-5; phospho-MET ELISA, R&D Systems DYC2480-5; HGF ELISA, R&D Systems SHG00). All the data presented were normalized to the total amounts of protein present in the extracts.

For immunofluorescence analysis(IF) assay, cells grown on glass slides were fixed in 4% paraformaldehyde for 15 minutes. Subsequently, the cells were permeabilized with 0.1% Triton X-100 for 15min and then blocked with PBS containing 5%(wt/vol) bovine serum albumin for 1 hour at room temperature. Cells were stained with primary antibodies for 12 hours at 4°C. The cells were washed with PBS, incubated with Alexa Fluor 488/594–conjugated goat anti-mouse/rabbit antibody (Santa Cruz Biotechnology, CA, USA), and counterstained with DAPI. Fluorescence signal was then visualized using fluorescence microscopy (Leica Microsystems Imaging Solutions, Cambridge, UK).

**RNA isolation and real-time quantitative reverse-transcription PCR**

Total RNA was isolated using Trizol reagent (Invitrogen). A total of 2μg of high quality RNA was processed directly to cDNA with the reverse transcription kit (Promega), following the manufacturer’s instructions, in a total volume of 25μl. Amplification reactions were performed in a 15 μl volume of the LightCycler-DNA Master SYBR Green I mix (Roche Applied Science, Penzberg, Germany) with 10 pM of primer, 2 mM MgCl_2_, 200 μM deoxynucleotide triphosphate mixture, 0.5 units of Taq DNA polymerase and universal buffer. All reactions were performed in triplicates in Mx3000 system(Stratagene), and thermal cycling conditions were as follows: 95°C for 3 min; 40 cycles of 95°C for 30 s, 60°C for 20 s, and 72°C for 20 s; 72°C for 10 min. The following primer pairs were used:

MET-f/MET-r: AGCAATGGGGAGTGTAAAGAGG/ CCCAGTCTTGTACTCAGCAAC

GAPDH-f/GAPDH-r: AGCCACATCGCTCAGACAC/ GCCCAATACGACCAAATCC

Relative mRNA levels were calculated using the –△△Ct method with GAPDH as the control and expressed as 2^(–△△Ct).

**DNA mutation analysis**

Genomic DNA was extracted from the cancer cells with QIAamp DNA FFPE Tissue kit (Qiagen, CA, USA) according to the manufacturer’s instructions. The DNA concentration was determined with a NanoDrop 2000 (Thermo Fisher Scientific, Wilmington DE, USA). The extracted DNA with either low concentration or bad purity was excluded from the mutation analysis. Exon 14 of the MET gene was amplified with primer by Polymerase chain reaction (PCR). The primer sequence was as follow: forward primer: 5- TTTCCTGTGGCTGAAAAAGAGA-3, reverse primer: 5- TTGCTCAAATTAACCTTTTTGTGAA-3. The cycling conditions were as follows: initial denaturation at 95°C for 4 minutes followed by 35 cycles at 95°C for 50 seconds, at 60°C for 50 seconds, and at 72°C for 1 minute, and one cycle at 72°C for 10 minutes. The PCR products were purified, and direct sequencing was performed using ABI377 DNA Analyzer. Both forward and reverse sequencing reactions were carried out with the Genbank-archived human sequence for MET.

**Cell proliferation, colony-formation assays and capillary tube formation analysis**

Cell proliferation was assessed with the cell counting kit-8 (Dojindo, Kumamoto, Japan) in accordance with the manufacturer’s instructions. Briefly, HCC cells were seed onto 96-well plate for 24h. Then various concentrations of the compounds were added into the wells. After treatment for 48 h, 10μl CCK-8 reagent was added and incubated for an additional 2 h. The absorbance was read at 450 nm using a microplate reader (Thermo, MD, USA). The IC50 value (half maximal inhibitory concentration**)** was calculated by GraphPad Prism software (San Diego, CA, USA).

For colony-formation assay, a total of 1×10^3^ cells per well were plated in 6-well plates, and 24 h later, the cells were treated with different inhibitors. After 2 weeks, cells were fixed with 4% paraformaldehyde for 15 minutes and visualized by staining the cells with 0.5% crystal violet for 30 min.

For capillary tube formation analysis, HUVECs (5 × 10^4^ per well) were cultured at 37°C for 6 hours in a 24-well plate coated with Matrigel (BD Biosciences, Bedford, MA) in the absence or presence of VEGF (50 ng/mL). The formation of capillary-like structures were captured under a light microscope. The number of the formed tubes, which represent the degree of angiogenesis *in vitro*, were scanned and quantitated in five lowpower fields (200× magnification).

**Cell invasion and wound-healing assays**

Cell invasion assays were performed using 24-well transwell chambers coated with a thin layer of Matrigel Basement Membrane Matrix (BD Biosciences, Bedford, MA). In total, 5 × 10^4^ cells in serum free DMEM containing 0.1% DMSO or various concentrations of inhibitors were added to the upper chamber, and 0.6 ml of low serum (1% FBS) DMEM with 10 ng/ml of HGF was placed in the lower chamber. After 48 hours of incubation, cells that migrated to the underside of the membrane were stained with Giemsa (Sigma Chemical Company, Saint Louis, MO), imaged, and counted with a microscope (Leica, UK). The chemotactic index was calculated as the ratio of the number of cells that migrated to different amboceptor-containing wells divided by the number of cells that migrated to cultured medium alone.

For wound healing assay, the cellular monolayer was grown to confluence in 6-well plates, wounded by scratching with a pipette tip, and washed with PBS. Wounded cells were starved in DMEM containing 1% FBS in the presence of 10 ng/mL HGF or the combination of HGF and1uM NZ001 for 24 h. The wounds were photographed (10 × objective) after 48 h. Each experiment was performed in triplicate.

**Immunohistochemical analysis and diagnostic scoring system**

Harvested tumors or liver tissues were fixed in 4% formaldehyde solution in PBS, and embedded in paraffin. 5mm thick sections were cut from paraffin-embedded tissue blocks, deparaffinized and rehydrated in ethanol, and then subjected to antigen retrieval. Endogenous peroxidase activity was blocked using 0.3% hydrogen peroxide in methanol for 30 min. Sections were blocked with 2% BSA in PBS for 1h at 37 °C, followed by incubation with primary antibodies at 4°C overnight. After washing with PBS three times on the second day, corresponding secondary antibodies were applied, and samples were further incubated at 37 °C for 1h. Slides were visualized with DAB staining.

Immunohistochemical staining was assessed by 3 independent investigators who were blinded to tumors characteristics, and discrepancies were resolved by consensus. Under 200× magnification, photographs of 3 representative fields were captured by the Leica QWin Plus v3 software; identical settings were used for each photograph. Tumor cells in three fields were randomly selected and staining intensity was scored as 0=no staining, 1=weak staining, 2=moderate staining, and 3=strong staining. The percentage of positively stained cells were scored as 0=no staining, 1=0-25% staining, 2=25%-50% staining, 3=50%-75% staining and 4= 75%-100% staining. The final IHC score was calculated by multiplying the intensity score with the percentage score. Negative controls were obtained by omitting the primary antibody. For Ki-67, HIF-1α and cleaved-PARP, only nuclear immunoreactivity was considered positive. Quantification of CD34 staining in tumor tissues of xenografts was calculated as CD34 -positive area at 200×magnification in 5 spots of each section. The proliferation, apoptosis and HIF-1α score were corresponded to the number of labeled Ki-67, cleaved PARP cells or HIF-1α among at least 500 cells per region and were expressed as percentages. The apoptosis index was calculated using (At-Ct)×100%, where At is the apoptosis score of the treated group at indicated time t and Ct is apoptosis score of untreated mice at time t. The proliferation inhibiton index and MVD inhibition index was calculated using (Ct-Pt)×100%, where Pt is the proliferation score or CD34 staining of the treated group at indicated time t and Ct is proliferation score or CD34 staining of untreated mice at time t.

The MET/P-MET IHC diagnostic scoring system was used to evaluate both staining intensity (negative, weak, moderate, or strong) and prevalence of these intensities in tumor cells. The four MET diagnostic subgroups were defined as: 3+ (≥ 50% of tumor cells staining with strong intensity); 2+ (≥ 50% of tumor cells with moderate or higher staining but<50% with strong intensity); 1+(≥50% of tumor cells with weak or higher staining but < 50% with moderate or higher intensity); 0 (no staining or<50% of tumor cells with any intensity).

**Evaluation of *in vivo* tumor growth and metastasis in mice models of HCC**

Male BALB/c nu/nu mice and C57BL/6 mice (4–6 weeks old, Shanghai Institute of Material Medicine, Chinese Academy of Sciences) were housed in specific pathogen-free conditions. All animals were fed a standard diet ad libitum and housed in a temperature-controlled animal facility with a 12/12 hour light/dark cycle. All animals received humane care according to the criteria outlined in the “Guide for the Care and Use of Laboratory Animals” (NIH publication 86-23 revised 1985).

Subcutaneous implantation models were established using MHCC-97H and Huh7 cells. A 0.1 mL of cell suspension (5×10^7^/mL in PBS) was subcutaneously implanted into the right flank of mice and grew for 1 week to reach a tumor size of approximately 50 to 100 mm^3^. The models were then randomized into three groups: vehicle control (ddH_2_O, orally), NZ001 (10 mg/kg/d, orally), or NZ001 (30 mg/kg/d, orally). Tumor volume (mm^3^) was calculated by the following formula: ab^2^/2 (where a and b refer to the largest and smallest dimensions collected every 3 days after treatment). The mice were sacrificed after 14 days of treatment. The tumors were excised, weighed, and either processed for paraffin embedding or snap frozen and stored at -80°C. Tumor growth inhibition (TGI%) was calculated using {1-[(Tt/T0)/(Ct/C0)]/1-[C0/Ct]}×100, where Tt is the tumor volume of the treated group at indicated time t; T0 is the original tumor volume of the treated animal; Ct is median tumor volume of untreated mice at time t; and C0 is the median original tumor volume of the control group.

To establish patient-derived xenografts, primary tumor specimens were collected from HCC patients who underwent tumor resection at Huashan Hospital, Fudan University (Shanghai, China) in 2017. Six-week-old male BALB/c nude mice under pathogen-free conditions were used for patient derived xenograft transplantation. Briefly, primary HCC tumor samples were minced into 10mm^3^ sized fragments and injected directly into the right flank of mice. The tumor formation was monitored in the next three months since implantation. Patient derived xenografts from donor mice reaching 1000-2000 mm^3^ were aseptically excised and dissected into fragments of approximately 10 mm^3^ and transferred to culture medium before subcutaneous implantation into receiving mice. All tumor fragments were obtained from the same passage and all mice were implanted on the same day. The time from cancer samples collection to mouse implantation ranges from 30-180 min. Once the average tumor size reached approximately 50 to 100 mm^3^, the mice with *MET*-amplification or MET-overexpression tumors were randomized into two groups: vehicle control (ddH_2_O, orally) and NZ001 (30 mg/kg/d, orally). The mice were sacrificed after 14 days of treatment. The tumor volumes were evaluated as described above. In the survival observation groups, treatments were started one week after implantation of the tumors until they died.

To further evaluate their effects on the *in vivo* tumor growth and metastasis of HCC, we established the orthotopic implantation mice models using Hepa1-6 cells. Briefly, Hepal-6 cells were first subcutaneously inoculated into the right flanks of 4-6 weeks C57BL/6 male mice. After 3-4 weeks, non-necrotic tumor tissues were cut into 1mm^3^ pieces and orthotopically implanted into the liver. One week later, the established orthotopic models were randomly assigned into 4 groups (n= 4/group): vehicle control (ddH_2_O, orally); PF-04217903 group, orally administered 30 mg/kg every day; anti-VEGF Ab group, administered via the intraperitoneal injection with 7.5 mg/kg anti-VEGF Ab every other day; and combination group, administered 30 mg/kg PF-04217903 and 7.5 mg/kg anti-VEGF Ab. The mice were sacrificed after 14 days of treatment. Another 3 groups (n=5/group) were randomly assigned to receive a daily oral dose of vehicle solution, 30 mg/kg sorafenib or 30mg/kg NZ001 for 14 days.

To further evaluate the effects of NZ001 on the lung and liver metastasis of HCC, we established the experimental metastasis models using MHCC-97H cells. MHCC-97H cells (1× 10^6^ cells) in 0.1 mL PBS were injected directly into the tail veins of 4- to 5-week-old male nude mice. One week later, the established metastatic models were randomly assigned into 2 groups (n= 11/group): vehicle control (ddH_2_O, orally) and NZ001 group, orally administered 30 mg/kg every day. Mice were sacrificed after daily treatment with NZ001 for 21 days, and their lungs were weighed. To examine the metastases, 100 sequential sections (5 mm) were cut from the lungs and livers of each mouse, and every 10th section was stained with hematoxylin and eosin (H&E).
