# Supplementary material for: The dual blockade of MET and VEGFR2 signaling demonstrates pronounced inhibition on tumor growth and metastasis of hepatocellular carcinoma
Source: J Exp Clin Cancer Res. 2018 Apr 30;37:93. doi: 10.1186/s13046-018-0750-2 (PMC5925844; doi:10.1186/s13046-018-0750-2)
Supplement: Supplementary file 3 — Figure S1. The E-cadherin, vimentin, CD34, HIF-1α, P-MET and total-MET expression levels in hepa1–6 orthotopic tumors after treatment with vehicle and VEGF antibody. Figure S2. The expression levels of E-cadherin, N-cadherin and vimentin were detected by Western blot in HCC cells starved overnight and treated with HGF (10 ng/ml) for 24 h. Figure S3. After 2-week treatment with VEGF antibody, PF-04217903 alone, and their combination, the mice were killed and the liver tissues were obtained. Figure S4. The E-cadherin, vimentin, CD34, HIF-1α, total-MET and P-MET expression levels in hepa1–6 orthotopic tumors after treatment with VEGF antibody and PF-04217903 alone, or their combination. Figure S5. The effect of NZ001 on VEGFR2 and MET signaling in HUVECs and HCC cells. Figure S6. NZ001 suppressed the HGF-induced EMT in HCC cells. Figure S7. Effects of NZ001 on the spontaneous and chemokine-induced invasion of HCC cell under normoxia and hypoxia condition. Figure S8. After 2-week treatment with sorafenib and NZ001, the mice were killed and the liver tissues were obtained. Figure S9. The total-MET, HIF-1α, CD34 and P-MET expression levels in hepa1–6 orthotopic tumors after treatment with VEGF antibody and PF-04217903 alone, or their combination. Figure S10. Effects of NZ001 on the metastasis of MHCC97H in nude mice. Figure S11. Effects of NZ001 on colony-formation of HCC cells. Figure S12. The association of MET exon 14 mutation determined by sanger sequencing with NZ001 sensitivity of 12 HCC cell lines. Figure S13. The score of MET and P-MET definition for IHC staining. Figure S14. The MET/P-MET/HGF expression in HCC cell lines. Figure S15. After 2-week treatment with different concentrations of NZ001, the mice were sacrificed and the tumors were obtained. Figure S16. IHC assay showed that MET phosphorylation was greatly inhibited after NZ001 treatment in MHCC-97H xenograft tumors. (DOCX 5327 kb) [file 13046_2018_750_MOESM3_ESM.docx]

**Additional file 3**


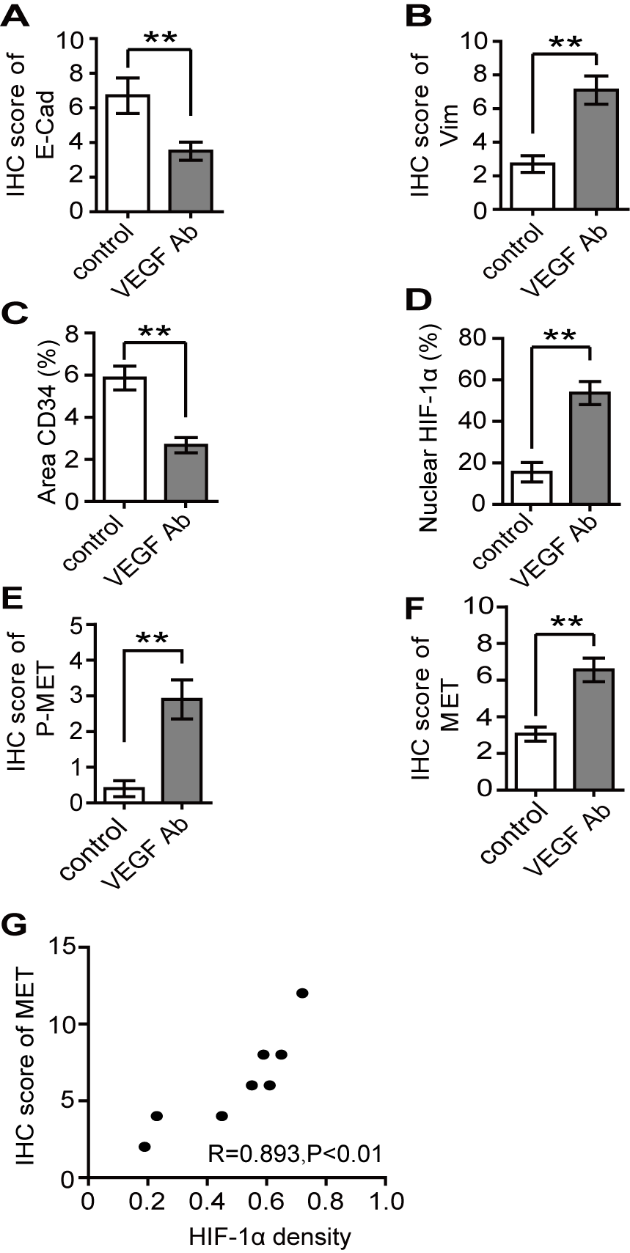


**Figure.S1. The E-cadherin, vimentin, CD34, HIF-1α, P-MET and total-MET expression levels in hepa1-6 orthotopic tumors after treatment with vehicle and VEGF antibody.**

**a-f** The IHC scores of E-cadherin, vimentin, CD34, HIF-1α, P-MET and total-MET in hepa1-6 orthotopic tumors after treatment with vehicle and VEGF antibody for 2 weeks. Significant differences were determined using Student’s *t* test.

**g** Scatter plot diagram based on immunohistochemistry analysis showed a significant positive correlation between HIF-1α and total-MET levels in tumor tissues treated with VEGF Ab. The correlation was analyzed by Pearson correlation analysis.

Data are shown as the mean ± SD. *: *P*<0.05; **: *P*<0.01; NS: No Significance.


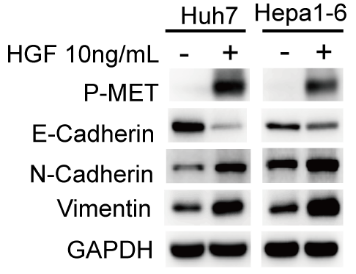


**Figure S2. The expression levels of E-cadherin, N-cadherin and vimentin were detected by Western blot in HCC cells starved overnight and treated with HGF (10ng/ml) for 24 hours.**


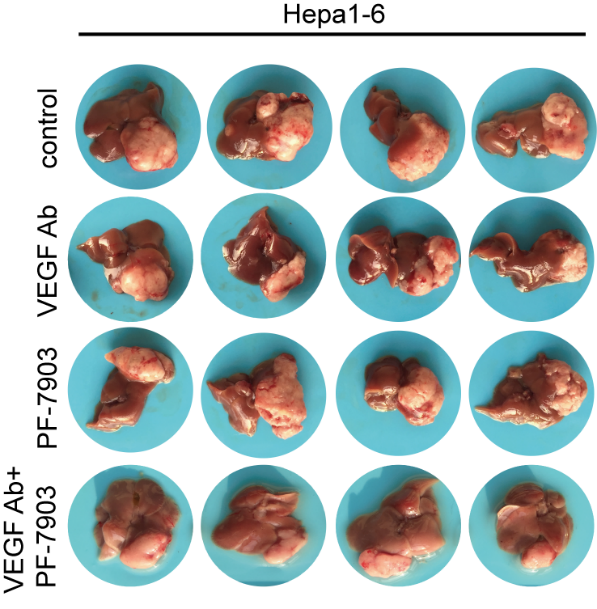


**Figure S3. After 2-week treatment with VEGF antibody, PF-04217903 alone, and their combination, the mice were killed and the liver tissues were obtained.**


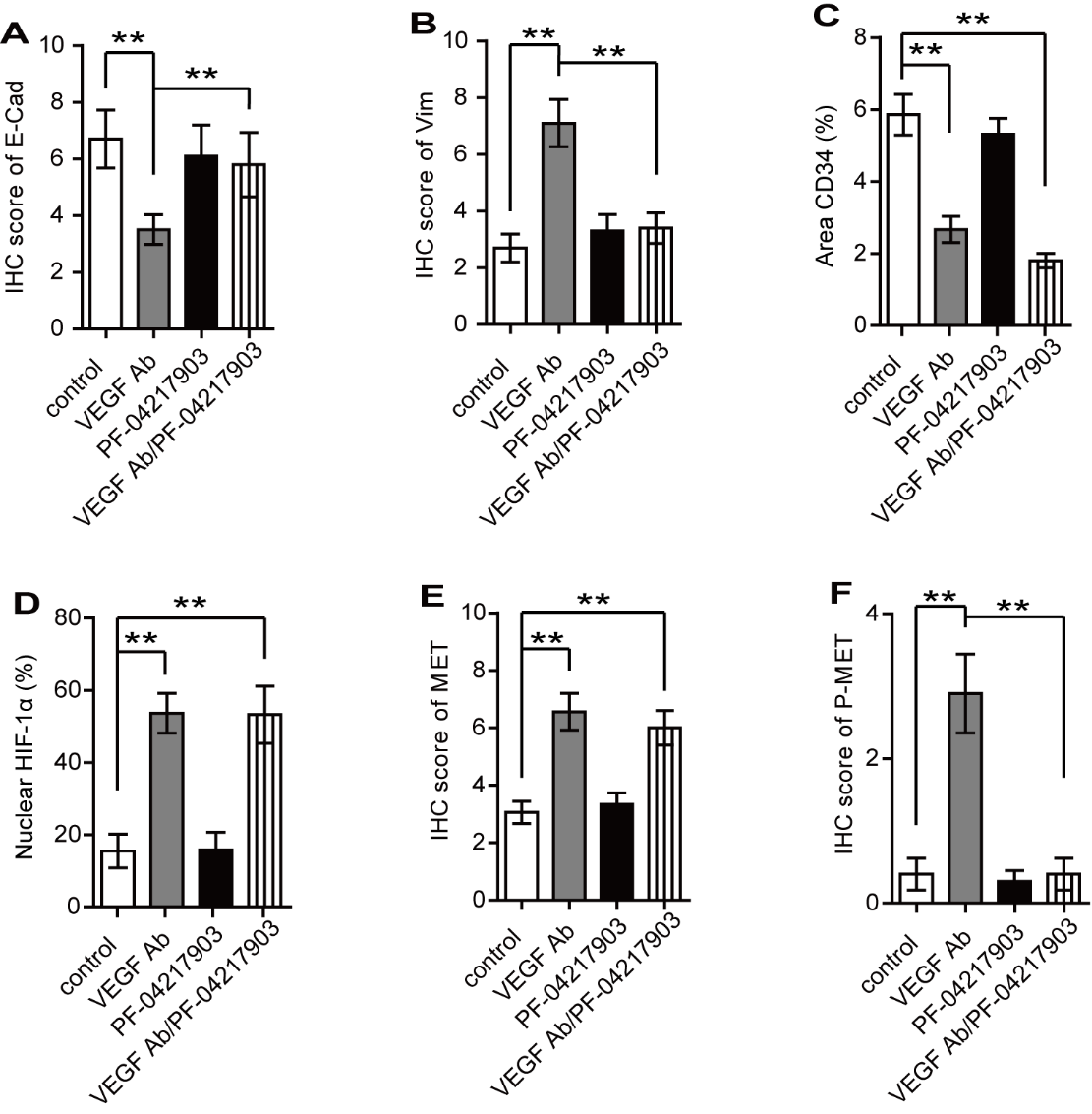


**Figure S4. The E-cadherin, vimentin, CD34, HIF-1α, total-MET and P-MET expression levels in hepa1-6 orthotopic tumors after treatment with VEGF antibody and PF-04217903 alone, or their combination.**

**a-f** The IHC scores of E-cadherin, vimentin, CD34, HIF-1α, P-MET and total-MET in hepa1-6 orthotopic tumors after treatment with VEGF antibody and PF-04217903 alone, or their combination for 2 weeks.

Data are shown as the mean ± SD. Significant differences were determined using one-way ANOVA. *: *P*<0.05; **: *P*<0.01; NS: No Significance.


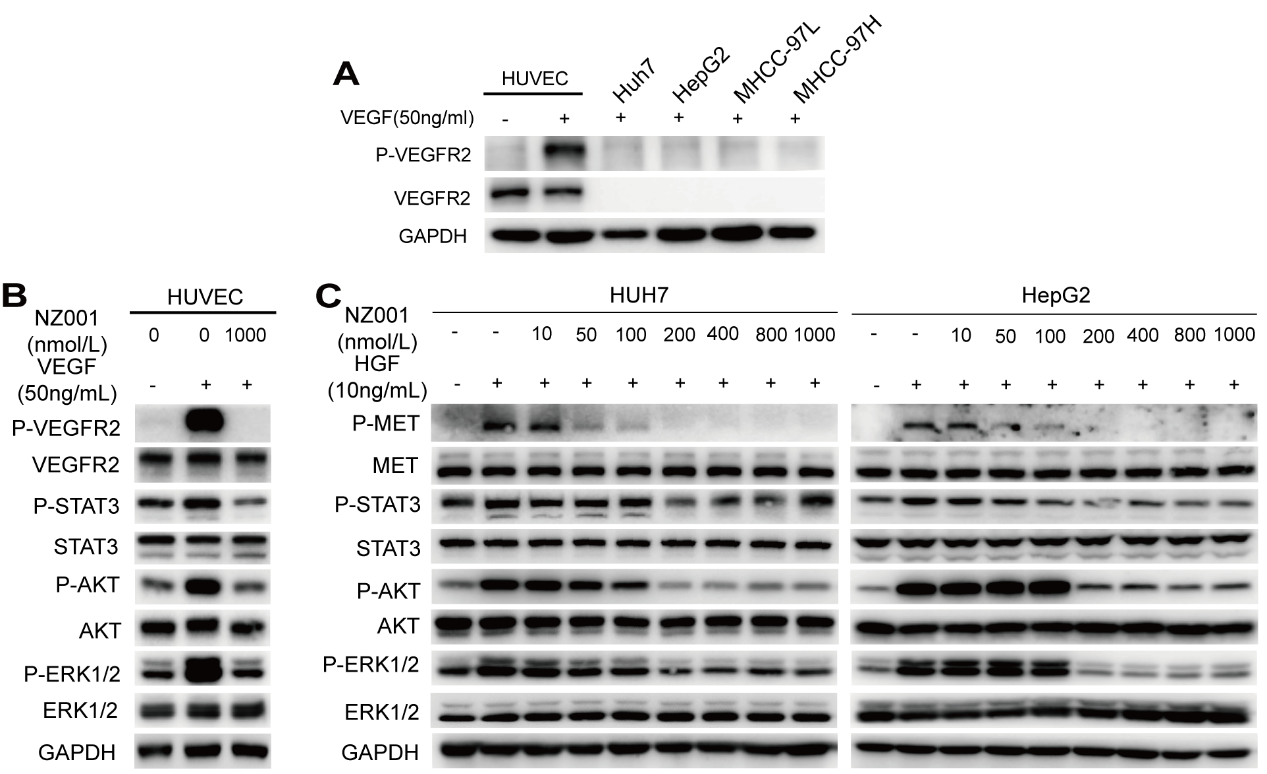


**Figure S5. The effect of NZ001 on VEGFR2 and MET signaling in HUVECs and HCC cells.**

**a** Western blot analysis was performed to detect the expression profile of VEGFR2 in HUVECs and HCC cells.

**b** NZ001 inhibited VEGF-induced phosphorylation of VEGFR2 and its downstream effectors STAT3, ERK1/2 and AKT in endothelial cells(HUVECs).

**c** NZ001 blocked HGF-induced phosphorylation of MET and its downstream effectors STAT3, ERK1/2 and AKT in HCC cells.


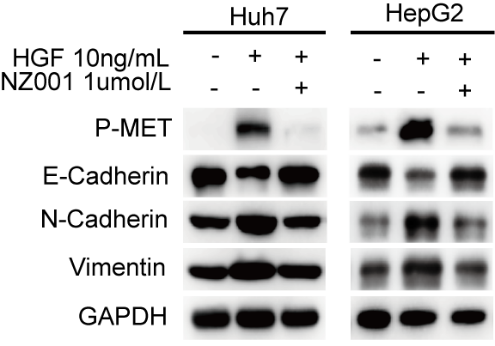


**Figure S6. NZ001 suppressed the HGF-induced EMT in HCC cells.**


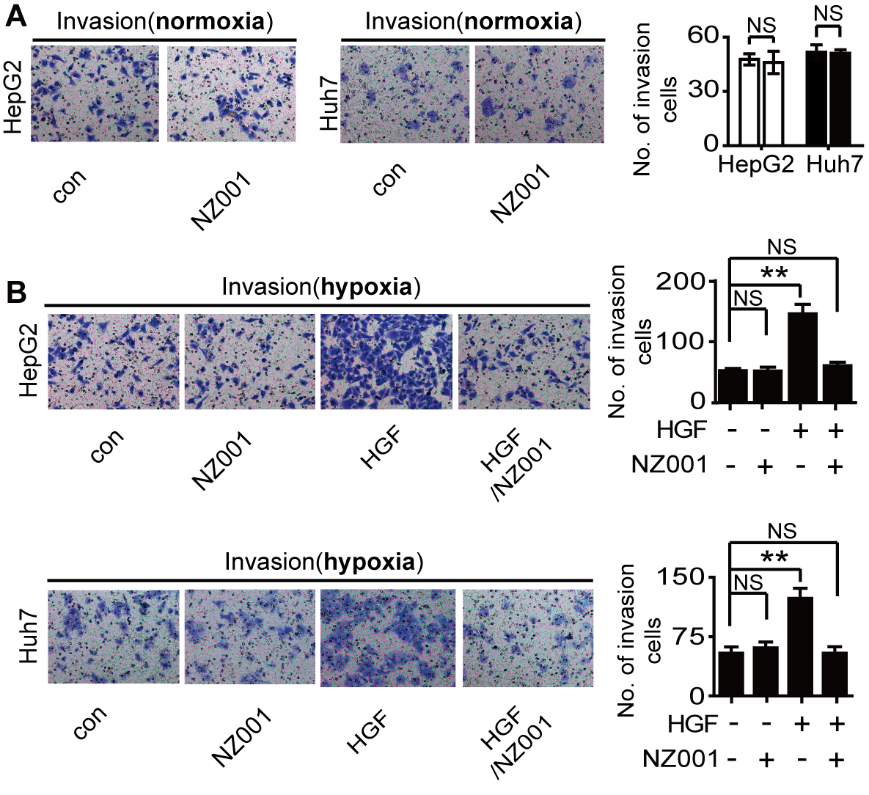


**Figure S7. Effects of NZ001 on the spontaneous and chemokine-induced invasion of HCC cell under normoxia and hypoxia condition.**

**a** NZ001(1umol/l) exert no significant inhibitory effect on spontaneous invasion of Huh7 and HepG2 cells in a Transwell assay.

b NZ001(1umol/l) exert no significant inhibitory effect on spontaneous invasion of Huh7 and HepG2 cells, but it significantly inhibited HGF(10ng/ml)-induced cell invasion of those cells under hypoxia condition in a Transwell assay.

Data are shown as the mean ± SD. Significant differences were determined using one-way ANOVA. *: *P*<0.05; **: *P*<0.01; NS: No Significance.


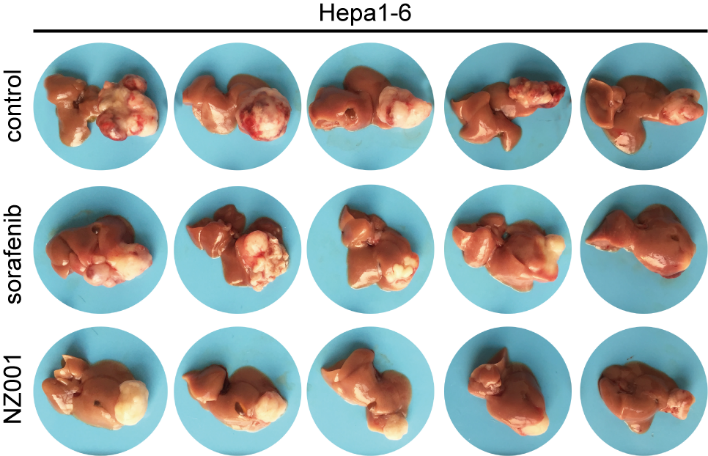


**Figure S8. After 2-week treatment with sorafenib and NZ001, the mice were killed and the liver tissues were obtained.**

**
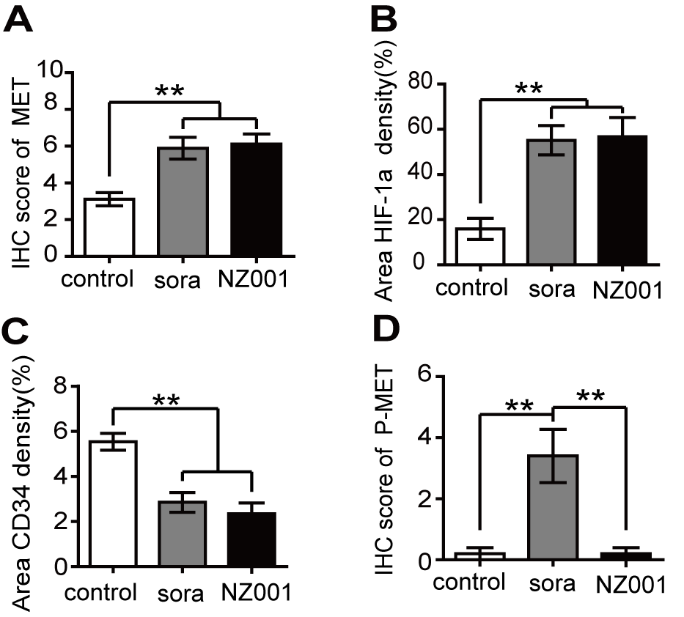
**

**Figure S9. The total-MET, HIF-1α, CD34 and P-MET expression levels in hepa1-6 orthotopic tumors after treatment with VEGF antibody and PF-04217903 alone, or their combination.**

**a-d** The IHC scores of total-MET, HIF-1α, CD34 and P-MET in hepa1-6 orthotopic tumors after treatment with NZ001 (30mg/kg/d) or sorafenib (30mg/kg/d) for 2 weeks.

Data are shown as the mean ± SD. Significant differences were determined using one-way ANOVA. *: *P*<0.05; **: *P*<0.01; NS: No Significance.

**
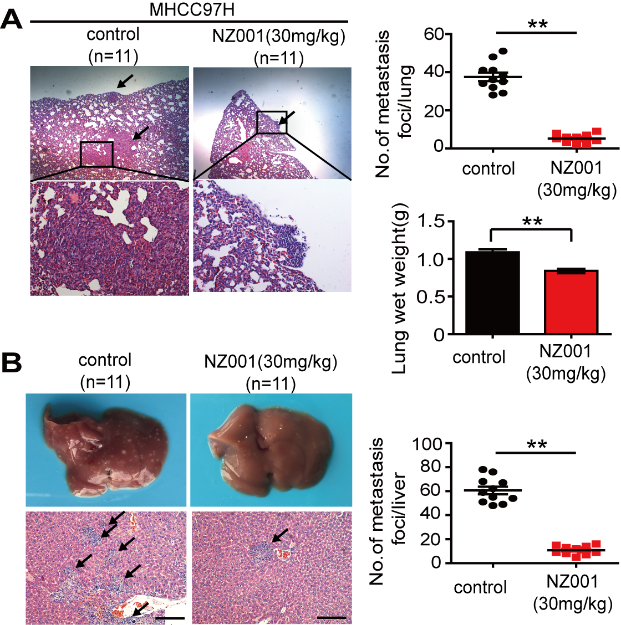
**

**Figure S10. Effects of NZ001 on the metastasis of MHCC97H in nude mice.**

**a-b** MHCC97H cells were directly injected into the tail vein of nude mice and the nude mice were sacrificed after 3 weeks treatment of NZ001 to evaluate the lung and liver metastases. The image is a representative HE-stained section of lung (**a**) and liver metastases (**b**).

Data are shown as the mean ± SD. Significant differences were determined using Student’s *t* test. *: *P*<0.05; **: *P*<0.01; NS: No Significance.


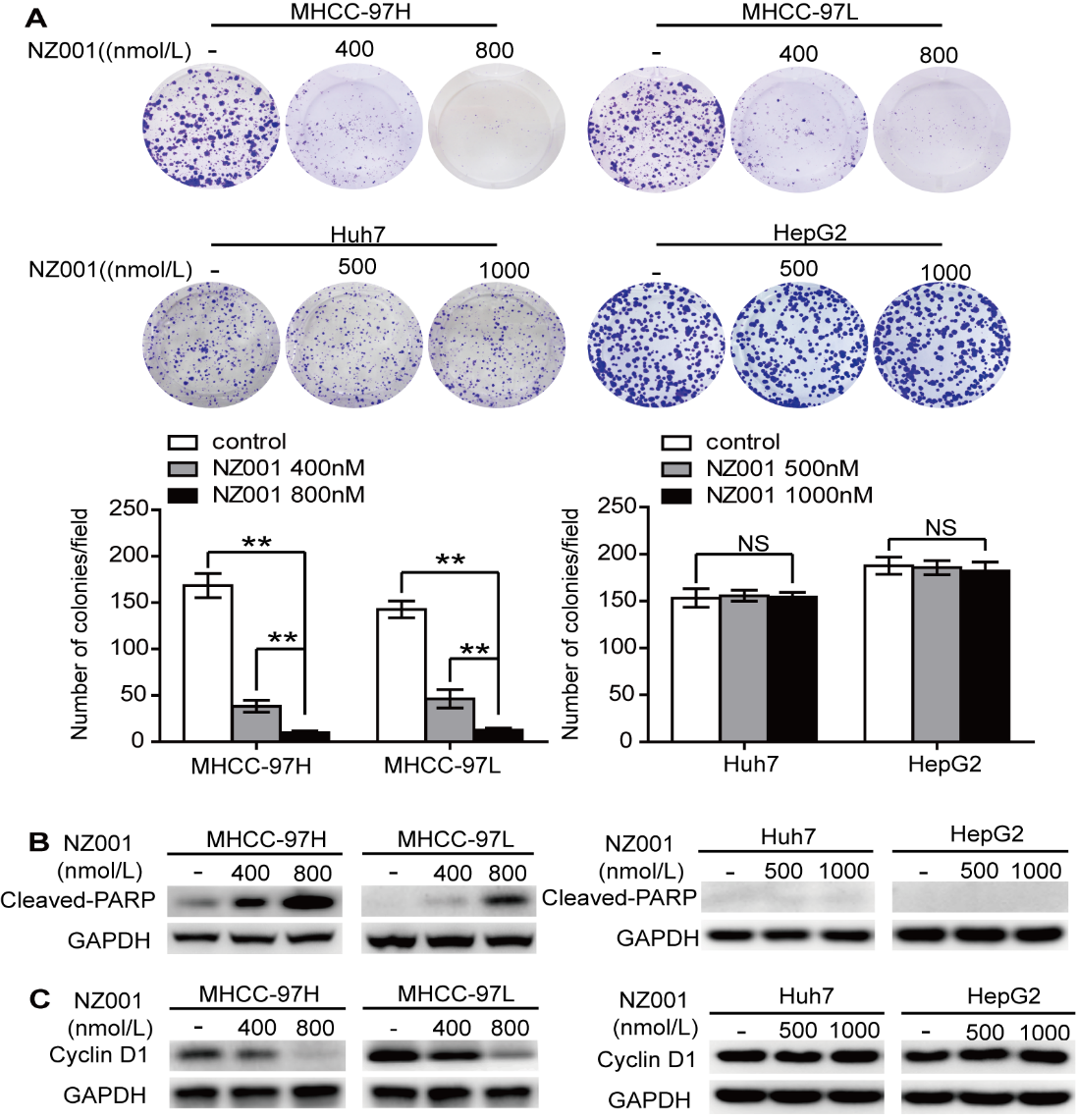
­

**Figure S11. Effects of NZ001 on colony-formation of HCC cells.**

**a** NZ001 blocked colony-formation of MHCC-97L and MHCC-97H cells.

**b-c** Western blot analysis was performed for cleaved-PARP (cell apoptosis marker) and Cyclin D1 (a critical regulator of the G1–S transition) expression in HCC cells treated with different concentrations of NZ001 for 24 hours.

Data are shown as the mean ± SD. Significant differences were determined using one-way ANOVA. *: *P*<0.05; **: *P*<0.01; NS: No Significance.


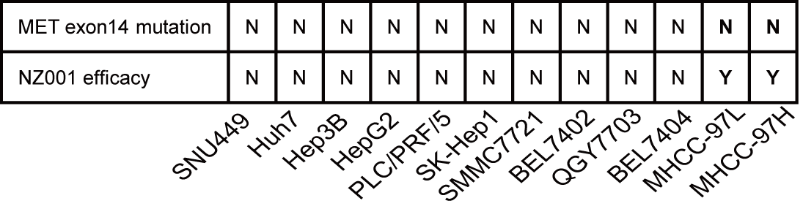


**Figure S12. The association of *MET* exon 14 mutation determined by sanger sequencing with NZ001 sensitivity of 12 HCC cell lines.**


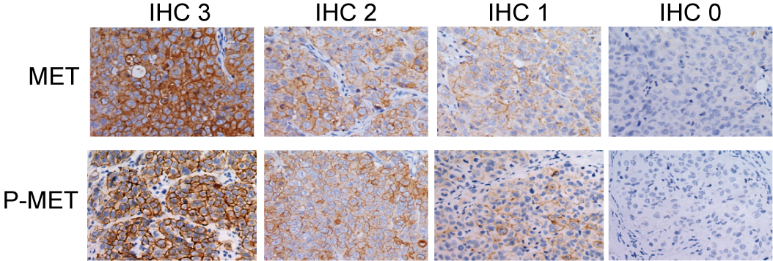


**Figure S13. The score of MET and P-MET definition for IHC staining.**

**
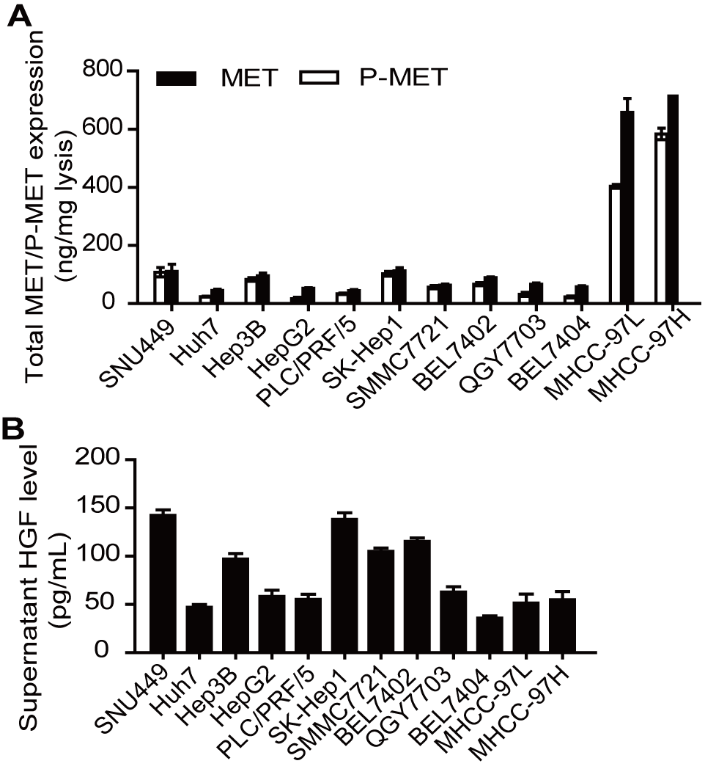
**

**Figure S14. The MET/P-MET/HGF expression in HCC cell lines.**

**a** The total-MET and P-MET protein levels in the 12 HCC cell lines determined by ELISA.

**b** The HGF levels in the culture supernatants of 12 HCC cell lines determined by ELISA.


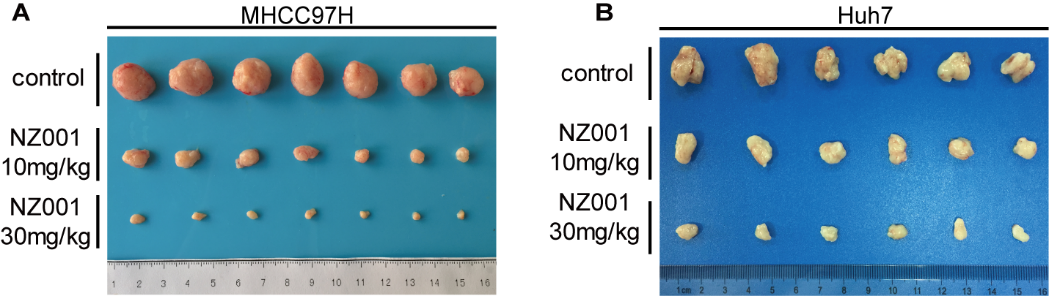


**Figure S15. After 2-week treatment with different concentrations of NZ001, the mice were sacrificed and the tumors were obtained.**


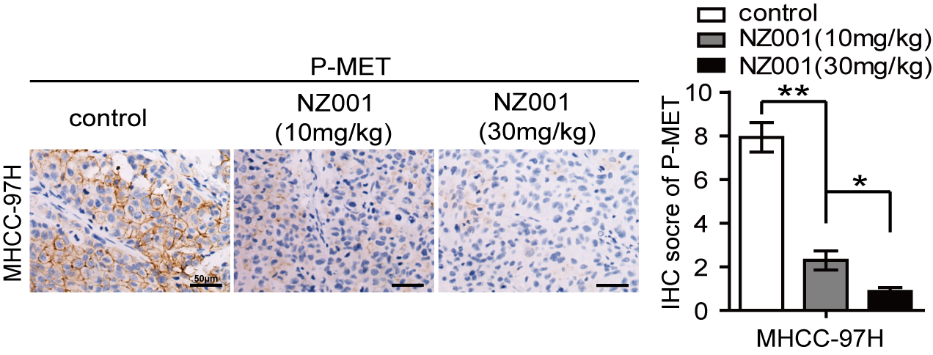


**Figure S16.** **IHC assay showed that MET phosphorylation was greatly inhibited after NZ001 treatment in MHCC-97H xenograft tumors.**

Data are shown as the mean ± SD. Significant differences were determined using one-way ANOVA. *: *P*<0.05; **: *P*<0.01; NS: No Significance.
